# Branched-chain amino acid supplementation and voluntary running have distinct effects on the serum metabolome of rats with high or low intrinsic aerobic capacity

**DOI:** 10.3389/fnut.2024.1450386

**Published:** 2024-11-19

**Authors:** Sira Karvinen, Tia-Marje K. Korhonen, Ronja Kiviö, Sanna Lensu, Bharat Gajera, Steven L. Britton, Lauren G. Koch, Anni I. Nieminen, Heikki Kainulainen

**Affiliations:** ^1^Gerontology Research Center and Faculty of Sport and Health Sciences, University of Jyväskylä, Jyväskylä, Finland; ^2^Department of Biological and Environmental Science, University of Jyväskylä, Jyväskylä, Finland; ^3^Faculty of Sport and Health Sciences, University of Jyväskylä, Jyväskylä, Finland; ^4^Department of Psychology, University of Jyväskylä, Jyväskylä, Finland; ^5^Metabolomics Unit, Institute for Molecular Medicine Finland (FIMM), University of Helsinki, Helsinki, Finland; ^6^Department of Molecular and Integrative Physiology, University of Michigan, Ann Arbor, MI, United States; ^7^Department of Anaesthesiology, University of Michigan, Ann Arbor, MI, United States; ^8^Department of Physiology and Pharmacology, College of Medicine and Life Sciences, The University of Toledo, Toledo, OH, United States

**Keywords:** valine, leucine, isoleucine, metabolism, exercise, diet

## Abstract

**Introduction:**

A growing body of literature associates branched-chain amino acid (BCAA) catabolism to increased fatty acid oxidation and better metabolic health. Hence, BCAA-rich diets may improve body composition and muscle protein synthesis. However, the role of individual characteristics such as a low aerobic fitness, a well-established risk factor for cardio-metabolic diseases, has not been studied.

**Methods:**

This study examined 64 female rats from the high-capacity runner (HCR) and low-capacity runner (LCR) rat model. Rats from each line (HCR or LCR) were divided into four groups; differing from diet (CTRL or BCAA) and from the opportunity to voluntarily run on a running wheel (NONRUNNER or RUNNER). Groups were matched for body mass and maximal running capacity within each line. We measured maximal running capacity and metabolism before and after the intervention of diet and voluntary running activity. After the end of the experiment, serum samples were collected for metabolome analysis.

**Results:**

We are the first to show that BCAA supplementation has a more pronounced impact on LCRs compared to HCRs. Specifically, in LCR rats, BCAA supplementation led to reduced daily voluntary running distance and an enrichment of serine metabolism in the serum metabolome. While voluntary running increased food intake and energy expenditure, its effects on the serum metabolome were minimal in HCRs.

**Conclusion:**

The present research highlights the benefit achieved by combining BCAA supplementation with running activity, especially in the LCR line. Importantly, our results underscore the interconnected role of BCAAs and fatty acid metabolism in promoting overall metabolic health.

## 1 Introduction

It is well-known that low aerobic capacity is a major risk factor for cardio-metabolic diseases and vice versa, high aerobic capacity predicts better health (1, 2). Increasing experimental evidence links branched-chain amino acid (BCAA) catabolism to increased fatty acid oxidation and better metabolic health (3, 4). BCAAs (valine, leucine, and isoleucine) are non-polar essential amino acids. Most of our daily requirement for BCAAs comes from dairy products (milk proteins), meat, fish, eggs, beans, nuts, and whole-grain products (5). Unlike other amino acids, BCAAs are not catabolized directly by the liver (6, 7). Due to this feature, digested BCAAs end up in the bloodstream, thus being readily available for skeletal muscles and other tissues. However, the liver oxidizes BCAAs after their conversion to oxo-ketoacids in other tissues. BCAAs are oxidized for energy production, and thus, BCAA supplementation may affect positively by sparing muscle glycogen stores during endurance exercise. In addition, leucine stimulates translation and subsequently, protein synthesis in skeletal muscle (8, 9). Adequate dietary intake of BCAA is known to have beneficial effects on body composition by increasing the release of fatty acids from adipocytes and thus decreasing fat mass (10). Therefore, BCAAs as well as BCAA-rich food and supplements such as whey protein are widely used in sports (11, 12). Hence, accumulating evidence indicates that BCAA-rich diets improve muscle protein synthesis, body composition, and perhaps also aerobic performance.

On the other hand, a strong association of obesity and insulin resistance with increased blood levels of BCAAs has been observed in human subjects (13–15). Several studies show that increased BCAA levels can be used as biomarkers of various cardiometabolic diseases and disturbed metabolic conditions (13, 14, 16). Unlike the ingestion of excess glucose and fatty acids which can be stored, amino acids including BCAAs are not converted to protein for later use, suggesting that excess BCAA intake is controlled through catabolic pathways (17). Furthermore, the clinical importance of efficient BCAA catabolism is demonstrated in patients with genetic disorders of BCAA metabolism causing severe neurological conditions (18, 19).

Our previous studies using omics analysis have shown that elevated long-term leisure-time physical activity is associated with a low serum BCAA level (20), increased muscle BCAA degradation (21), and further with improved body composition. Inherited high aerobic capacity is associated with a leaner phenotype and with improved signature of muscle BCAA catabolism (22). Acknowledging this, it may well be that one's aerobic capacity—whether inherited or acquired—is at least one of the main factors regulating optimal serum BCAA levels.

Koch and Britton have developed two rat lines by employing artificial selection for performance on a maximal treadmill running test: low-capacity runners (LCRs) and high-capacity runners (HCRs) (23). After 28 generations of selection these selected lines differed by >8-fold for intrinsic aerobic exercise capacity (24). Compared to HCRs, LCRs have high risk factors for metabolic syndrome (25), lower physical activity (26), lower response to training (27), a shorter life span (28, 29), lower cognition (30), and signs of non-alcoholic fatty liver disease (31). Our previous study showed that HCRs have skeletal muscles with enriched expression of genes related e.g., to oxidative phosphorylation (OXPHOS), fatty acid metabolism, and BCAA metabolism (22). Furthermore, Overmyer et al. (32) showed using metabolomic and proteomic profiling that HCRs oxidize more efficiently fatty acids and BCAAs. Also, our previous metabolomic study indicated increased leucine catabolism in HCRs (33).

The aims of this study were to investigate using a metabolomic approach, dietary BCAA supplementation and/or voluntary running effects on metabolism and whether the metabolic changes are different in rats with high or low intrinsic aerobic capacity. Our results align with previous studies demonstrating that serum metabolome profiles can distinguish between HCR and LCR rat lines. Notably, we are the first to highlight that BCAA supplementation has a more pronounced impact on LCRs compared to HCRs. Specifically, in LCR rats, BCAA supplementation led to a reduced daily voluntary running distance and an enrichment of serine metabolism in the serum metabolome. Interestingly, while voluntary running increased food intake and energy expenditure, its effects on the serum metabolome were minimal. Our findings underscore the synergistic effects of combining BCAA supplementation with voluntary running, which were more significant than either intervention alone. Overall, our results emphasize the interconnected role of BCAAs and fatty acid metabolism in promoting metabolic health.

## 2 Materials and methods

### 2.1 Rat lines

**Figure 1:**
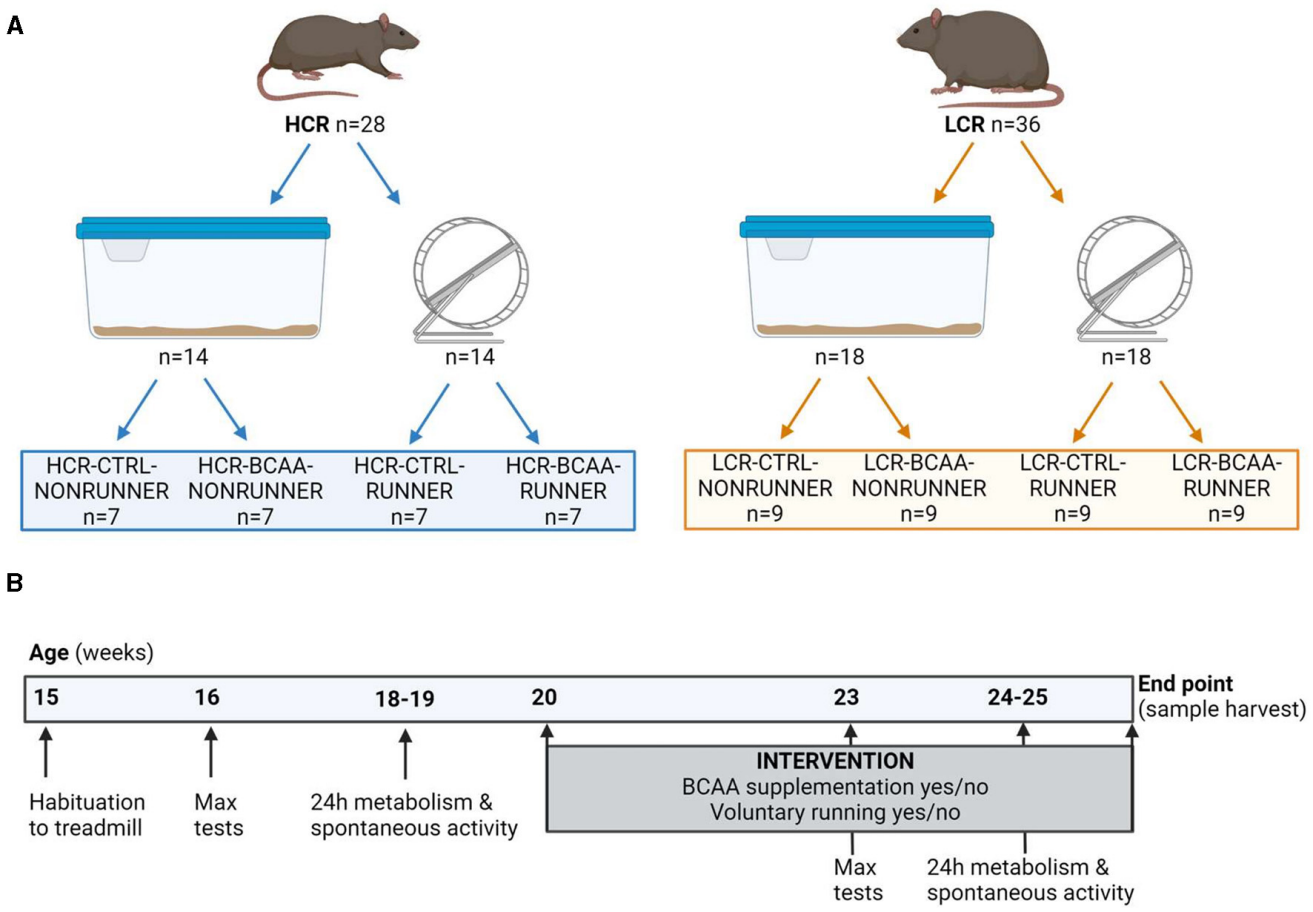
Schematic representation of the study setup **(A)** and study outline **(B)**. HCR, High-capacity runner; LCR, Low-capacity runner; CTRL, control diet; BCAA, Branched-chain amino acid diet; max tests, maximal running capacity tests. Created with BioRender.com.

The HCR/LCR contrasting rat model system utilized in the this study was produced via two-way artificial selection, starting from a large founder population (N:NIH stock) of genetically heterogeneous rats as described previously (23). Rats were bred onsite at the University of Jyväskylä from a founder population obtained from The University of Toledo (Toledo, Ohio, USA). For the present study 64 female rats (28 HCRs and 36 LCRs) produced from generations 42–43 of selection were used (Figure 1A). All rats were housed in an environmentally controlled facility (12/12 h light-dark cycle, 22°C) and received water and standard rodent feed (R36, Labfor, Stockholm, Sweden) *ad libitum*.

### 2.2 Ethical approval

The animal experiment was approved by the national Project Authorization Board (ELLA, Finland, permit number ESAVI-12840-2019). All procedures with the animals were conducted in accordance with the “Principles of Laboratory Animal Care” (NIH publication #85–23, revised in 1985) and the European Commission Directive 2010/63/EU. All efforts were made to minimize the number of animals used and their suffering.

### 2.3 Study design

Before the start of the intervention, rats were tested for their maximal running capacity with a speed-ramped treadmill running test (15° slope, initial velocity of 10 m min^−1^, increased 1 m min every 2 min) at the age of 16 weeks as described previously (23). Rats were first habituated to run on the treadmill with three different sessions lasting for 10 min with a low velocity (< 10 m min^−1^). Maximal running test was repeated three times with at least 1 day of recovery in between. The best result of the three trials [maximal running distance (m)] was considered the maximal running capacity. At the age of 18–19 weeks metabolism together with spontaneous activity were measured.

While HCR and LCR rat lines were bred based on maximal running capacity, the models also differs for body mass (23). Therefore, groups were matched within rat line according to maximal running capacity and body mass. Rats were divided into eight different study groups that differed in the rat line (HCR or LCR), BCAA supplementation (CTRL or BCAA), and opportunity to voluntarily run on a running wheel (NONRUNNER or RUNNER). In the HCR line, the number of animals (n) was seven per group and in the LCR line, n was nine per group (Figure 1A).

Age at the start of the intervention consisting of BCAA supplementation (yes/no) and voluntary running in a running wheel (yes/no) was 20 weeks (±2 weeks). After 3 weeks of intervention, the maximal running capacity (at the age of 23 weeks) and metabolism together with spontaneous activity (at the age of 24–25 weeks) were repeated as follow-up measurements (Figure 1B).

### 2.4 BCAA supplementation

BCAA supplementation was administered through drinking water. That is, during the BCAA supplementation period, rats in BCAA groups had only BCAA-supplemented drinking water available (supplemented with L-valine, V0513; L-leucine; L8912 and L-isoleucine, I7403, Sigma-Aldrich) with the following ratio: valine:leucine:isoleucine, 1.2:2:1 according to previous literature (34–36). We aimed to have two times higher (2x) BCAA intake for the BCAA-supplementation group compared to corresponding control group, as utilized previously (37, 38).

The target BCAA concentration of the water was determined for each BCAA-supplementation rat group in 2–3-day intervals. First, the average daily BCAA intake (mg) from feed of the corresponding control groups from the previous 2–3 days was determined. Second, the average daily BCAA intake (mg) from feed of the BCAA-supplementation groups from the previous 2–3 days was determined to calculate the BCAA amount needed to have 2xBCAA supplementation. Third, the average daily consumption of water (ml) of BCAA-supplementation group was measured from the previous 2–3 days to estimate the future consumption of water (see equation below).


$$\begin{array}{c} Water\;BCAA\;concentration\;\left(\frac{mg}{ml}\right)=\\ \frac{\begin{array}{c} 2\;x\;average\;daily\;BCAA\;intake\;from\;feed\;of\;corresponding\\ control\;group\;\left(mg\right)-average\;daily\;BCAA\;intake\;fromfeed\\ of\;the\;BCAA\;group\;\left(mg\right) \end{array}}{average\;daily\;water\;drinking\;volume\;\left(ml\right)} \end{array}$$

Thereafter, BCAA-content of the water was calculated separately for each BCAA-supplementation group depending on the average daily BCAA consumption of the corresponding control group and the average daily water consumption of each BCAA-supplementation group in 2–3-day intervals. Fresh BCAA-supplemented water was prepared from 50 mg/ml BCAA stock solution having the above-mentioned ratio of BCAAs. The BCAA-supplemented water was changed three times a week.

### 2.5 Body mass, energy intake, and voluntary running distance

Body mass and energy intake of the rats were followed throughout the intervention by weighing the rats and the food three times a week. The energy intake was calculated from the feed energy content information provided by the manufacturer (R36, Labfor, Stockholm, Sweden; 3.009 kcal/g). Voluntary running distance from the running wheels was followed with a computerized recording system as described earlier (39). Briefly, rats had the access to voluntarily run on a running wheel that was mounted on a cage (Techniplast 2154F0105, Buguggiate, Italy) with a custom-made software with data stored automatically to a server (MS SQL-server 2014 Express) (40). The total running distance per day was determined by multiplying the number of wheel rotations by the circumference of the running wheel (Ø 34.5 cm).

### 2.6 Metabolism and spontaneous activity

Metabolism was assessed via measurements from respiratory gases analyzing oxygen consumption [V[O_2_]] and CO_2_ production [V[CO_2_]] to obtain indirect measures of metabolism (Promethion^®^GA3, Sable Systems, Las Vegas, NV, USA) similarly as described previously (41). Briefly, the incurrent flow rate was set at 3,000 mL/min and the raw data were processed with ExpeData^®^ software (Sable Systems). Each rat was measured for 24 h before and after the intervention. Parameters V(O_2_), V(CO_2_), calorie consumption (kcal/h) and respiratory quotient (RQ, CO_2out_/O_2in_) were determined for the whole day (24 h) and separately for dark (12 h) and light (12 h) time of the day, since rats are active at dark time. V(O_2_) and V(CO_2_) were normalized to body mass (g).

Similarly, spontaneous activity was measured before and after the intervention from the same 24 h period. For this purpose, we used ground reaction force recordings, as described previously (42). To obtain a single value for total spontaneous activity, the 1-s means were summed for the total measurement time and the sum was divided by the body mass (kg) of the measured rat. Activity index was calculated for the whole day (24 h) and separately for dark (12 h) and light (12 h) time of the day.

### 2.7 Tissue harvest and tissue masses

At the end of the intervention, rats were euthanized after an overnight fasting using a mixture of air and carbon carbon dioxide inhalation followed by cardiac puncture. Rats in the BCAA-supplementation groups had BCAA water available until euthanasia. Tissues (heart, liver, and skeletal muscles) were weighed, snap frozen in liquid nitrogen and stored in −80°C for further future analysis. Serum was separated from the whole blood via centrifugation after 15 min incubation at RT (1,500 *g*, 10 min at RT) and stored as 200 μl aliquots in −80°C.

### 2.8 Metabolomic methods

Metabolites were extracted from 100 μl rat serum using 400 μl of cold extraction solvent (Acetonitrile:Methanol:Milli-Q Water; 40:40:20 v/v/v). Subsequently, the samples were vortexed for 2 min followed by centrifugation at 18.8^*^g and 4°C for 5 min. Supernatants were loaded into a Phenomenex, Phree Phospholipid removal 96 well plate 30 mg (Part No. 8E-S133-TGB) and passed through using robotic vacuum. Filtrates were transferred into HPLC auto sampler glass vials. For relative metabolomics profiling, 2 μl of samples were injected into Thermo Vanquish UHPLC coupled with Q-Exactive Orbitrap quadrupole mass spectrometer equipped with a heated electrospray ionization (H-ESI) source probe (Thermo Fischer Scientific). A SeQuant ZIC-pHILIC (2.1 × 100 mm, 5-μm particle) column (Merck) used for chromatographic separation. The gradient elution was carried out with a flow rate of 0.100 mL/min using 20 mM ammonium hydrogen carbonate in 100% water, adjusted to pH 9.4 with ammonium solution (25%) as mobile phase A and acetonitrile 100% as mobile phase B. The gradient elution was initiated from 20% Mobile phase A and 80% of mobile phase B and maintain till 2 min, after that 20% Mobile phase A gradually increase up to 80% till 17 min, then reversed to initial condition at 17.1 min and maintained up to 24 min. The column oven and auto-sampler temperatures were set to 40 ± 3 and 5 ± 3°C, respectively. The mass spectrometer was equipped with a heated electrospray ionization (H-ESI) source using polarity switching and following setting: resolution of 35,000, the spray voltages: 4,250 V for positive and 3,250 V for negative mode, the sheath gas: 25 arbitrary units (AU), and the auxiliary gas: 15 AU, sweep gas flow 0, Capillary temperature: 275°C, S-lens RF level: 50.0. Instrument control operated with the Xcalibur 4.1.31.9 software (M/s Thermo Fischer Scientific, Waltham, MA, USA). Metabolomics data processing and integration was performed with the TraceFinder 4.1 software (Thermo Fischer Scientific) using confirmed retention times from 463 metabolite standards (MSMLS-1EA, Merck). The data quality was monitored throughout the run using in-house serum sample as Quality Control (QC) interspersed throughout the run after every 10th sample. The detected metabolites were checked for peak quality (poor chromatograph), QC % RSD (20% cutoff) and background (20% cutoff).

### 2.9 Metaboanalyst analyses

MetaboAnalyst version 5.0 online tool (metaboanalyst.ca) was used for the analyses (43–46). For each analysis, the data was first logarithm 10 transformed and the autoscaling option was selected to mean-center the data and divide by the standard deviation of each variable. Partial Least Squares Discriminant Analysis (PLS-DA) and analysis of metabolite relative abundance were done with the Statistical Analysis (one factor) module using *t*-test *p*-value ≤ 0.050 and Fold change ≥1. Metabolite Set Enrichment Analysis (MSEA) was done with the Enrichment Analysis Module. For the Enrichment Analysis, the Quantitative Enrichment Analysis option was chosen. Metabolite sets based on KEGG pathways was selected.

### 2.10 Statistics

Data in tables and figures is presented as mean with SD. The normality of variables was assessed using Shapiro-Wilks tests followed by Levene's test for examining the equality of the variances. Body mass and maximal running capacity within group was assessed using One Way ANOVA to investigate differences between groups within a rat line. Paired samples *T*-test or Wilcoxon signed ranks test were used to investigate the difference between PRE and POST measurements. Differences between study groups were compared using Independent samples *T*-test or Mann-Whitney *U*-test. The main effects and interactions between rat line, BCAA and running were investigated using General linear model. When examining the effect of line in POST measurements, BCAA supplementation and running were added as covariates. One HCR was excluded from the PRE vs. POST maximal running capacity analysis due to unwillingness to run. Data analyses were carried out using IBM SPSS Statistics software version 24 (Chicago, IL, US), and the level of significance was set at *p* ≤ 0.050. In the analysis of serum metabolites and enriched metabolic pathways the FDR-adjusted *p*-value was utilized.

## 3 Results

**Figure 2:**
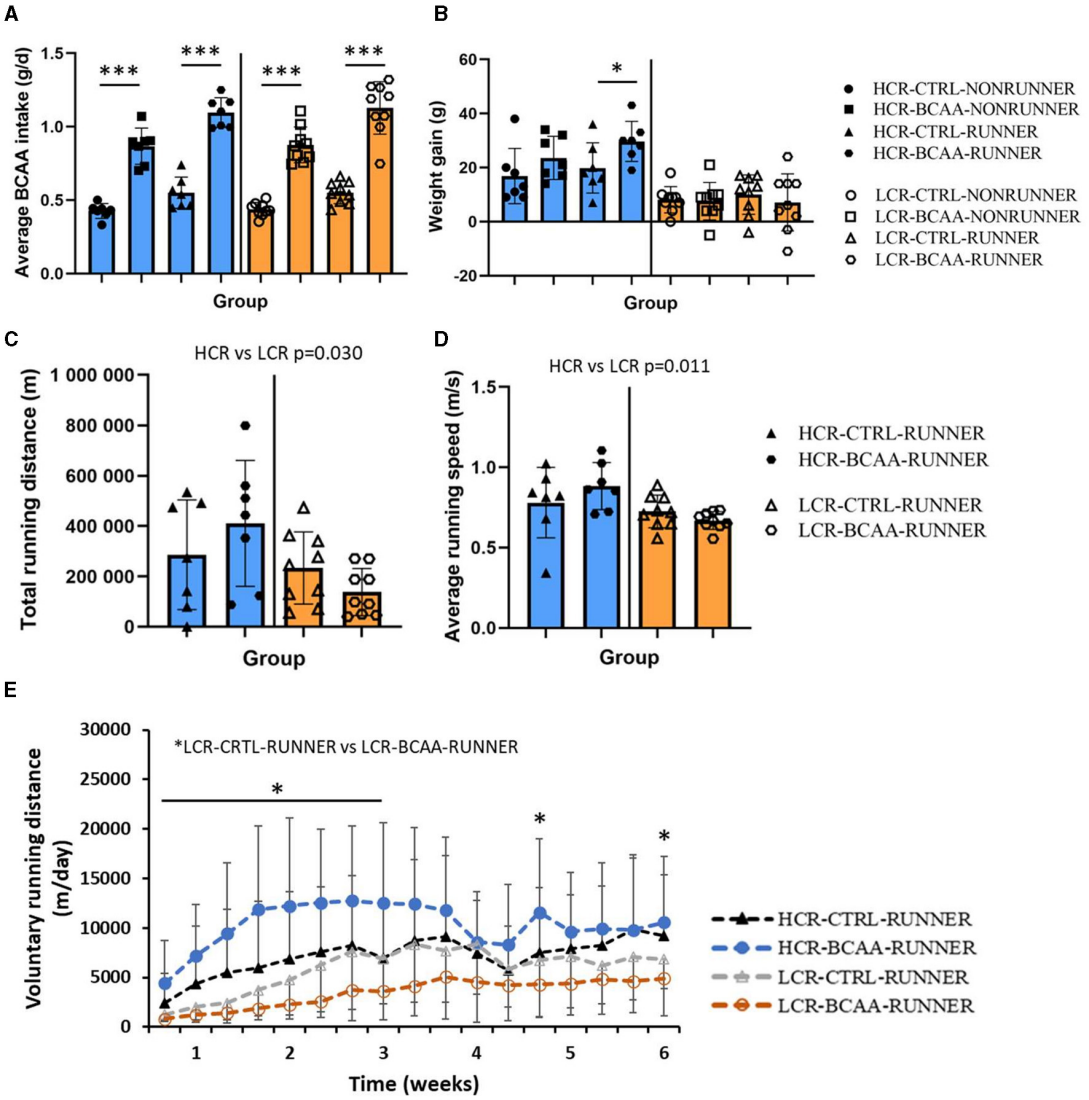
Average BCAA intake (g/d) **(A)**, body weight gain **(B)**, average total running distance (m) **(C)**, running speed (m/s) **(D)**, and voluntary running distance (m/day) **(E)** during the intervention. HCR, High-capacity runner; LCR, Low-capacity runner; CTRL, control diet; BCAA, Branched-chain amino acid diet. Data is presented as individual data points with mean and SD. *p ≤ 0.010, ***p ≤ 0.001, *p ≤ 0.050.

The successful BCAA intake was determined by measuring the BCAA intake from feed (all study groups) and BCAA-supplemented water (BCAA groups). As intended, BCAA groups had 2x higher BCAA intake compared to the corresponding control group (*p* ≤ 0.050, Figure 2A). When examining the body weight gain, HCR-BCAA-RUNNER group had a higher body weight gain compared to corresponding control group (POST-PRE, *p* < 0.050, Figure 2B). The detailed BCAA intake, body mass and food intake during the intervention are presented in Supplementary Figure 1. In HCRs, food intake was higher in RUNNER groups compared with NONRUNNER groups in all except the first two time points (*p* ≤ 0.013, Supplementary Figure 1E).

### 3.1 BCAA supplementation decreased daily voluntary running distance in LCRs

HCRs ran voluntarily more than LCRs and had a higher average running speed (*p* ≤ 0.011, Figures 2C, D). BCAA-supplementation had no significant effect on total running distance or average running speed. However, in LCRs, rats in LCR-CTRL-RUNNER group ran voluntarily more than rats in LCR-BCAA-RUNNER group during first 3 weeks and at one time point at the beginning of weeks 5 and 6 of intervention (*p* ≤ 0.050, Figure 2E).

Groups did not differ in body mass or maximal running distance within the rat line before the intervention (*p* ≥ 0.967, Table 1). After the intervention there was no difference in the body mass within rat line, even though all rats gained weight (*p* ≥ 0.846, Table 1), whereas maximal running capacity differed between the groups within rat line (*p* ≤ 0.002, Table 1). In HCR line, the maximal running capacity in the NONRUNNER groups did not change (*p* = 0.499, Table 1), but did increase in RUNNER groups (*p* ≤ 0.028, Table 1). Interestingly, in LCR line the maximal running capacity increased in all study groups (*p* ≤ 0.046, Table 1).

**Table 1:** Body mass and maximal running capacity (m) before and after intervention in the study groups (mean with SD).

	**Body mass (g)**			**Max running capacity (m)**		
	**PRE**	**POST**	***p***	**PRE**	**POST**	***p***
HCR-CTRL-NONRUNNER	194 (24)	220 (29)	**0.001**	2018 (518)	1839 (324)	0.499
HCR-BCAA-NONRUNNER	197 (24)	224 (17)	**< 0.001**	2048 (751)	1722 (621)	0.176
HCR-CTRL-RUNNER	193 (16)	218 (22)	**< 0.001**	2100 (358)	4009 (1429)	**0.028**
HCR-BCAA-RUNNER	191 (26)	225 (23)	**< 0.001**	2024 (618)	3656 (558)	**0.018**
LCR-CTRL-NONRUNNER	238 (16)	257 (23)	**0.002**	138 (53)	202 (58)	**0.028**
LCR-BCAA-NONRUNNER	238 (12)	254 (14)	**< 0.001**	137 (72)	217 (70)	**0.028**
LCR-CTRL-RUNNER	239 (19)	260 (17)	**< 0.001**	144 (75)	541 (386)	**0.008**
LCR-BCAA-RUNNER	236 (19)	252 (19)	**< 0.001**	140 (53)	482 (163)	**0.008**

p-values are from paired tests. Statistically significant findings marked in bold.

### 3.2 Voluntary running combined with BCAA supplementation was associated with higher respiratory quotient and energy expenditure

Before the intervention (PRE), HCRs had higher O_2_ consumption, lower CO_2_ production and higher spontaneous activity than LCRs (effect of line *p* ≤ 0.006, Table 2). Same was true for POST measurements before including covariates in the multivariate model (*p* < 0.001, *data not shown*). HCRs had also lower energy consumption PRE intervention during periods 24 h and dark, and lower respiratory quotient (RQ) during dark time (*p* ≤ 0.040, Table 2). After the intervention with covariates included (BCAA supplementation and running), HCRs had lower CO_2_ production (*p* = 0.048) and higher O_2_ consumption during darkness, which was close to being significant (*p* = 0.079, Table 2).

**Table 2:** Metabolism and spontaneous activity of the studied groups (mean with SD).

	**Group**								***p*** **-value**						
	**HCR-CTRL-NONRUNNER**	**HCR-BCAA-NONRUNNER**	**HCR-CTRL-RUNNER**	**HCR-BCAA-RUNNER**	**LCR-CTRL-NONRUNNER**	**LCR-BCAA-NONRUNNER**	**LCR-CTRL-RUNNER**	**LCR-BCAA-RUNNER**	**Line**	**BCAA**	**Run-ning**	Line^*^BCAA	Line^*^run-ning	BCAA^*^run-ning	Line^*^BCAA^*^running
**O**_2_ **consumption (ml/min/kg)**															
**Pre**															
24 h	27.797 (1.965)	27.235 (1.618)	27.450 (1.990)	28.897 (1.510)	23.234 (1.545)	23.993 (1.535)	24.119 (1.805)	23.030 (1.100)	**< 0.001**						
Dark (12 h)	31.056 (2.505)	30.262 (2.370)	29.961 (2.836)	31.817 (2.355)	25.226 (2.268)	26.863 (2.617)	27.241 (1.999)	25.603 (1.957)	**< 0.001**						
Light (12 h)	24.542 (1.565)	24.210 (1.419)	24.943 (1.765)	25.981 (0.919)	21.265 (1.142)	21.122 (1.073)	21.014 (1.786)	20.478 (0.434)	**< 0.001**						
**Post**															
24 h	25.561 (1.568)^*^	25.375 (1.049)^*^	27.371 (1.132)	27.911 (1.193)	22.034 (0.781)	22.615 (1.658)^*^	24.019 (1.488)	24.799 (1.421)^*^	0.14	0.204	**< 0.001**	0.454	0.895	0.492	0.696
Dark (12 h)	28.998 (1.607)^*^	29.040 (2.393)	31.769 (1.140)	31.887 (1.700)	24.061 (1.086)	25.579 (2.156)	27.979 (2.428)	28.276 (2.419)^*^	*0.079*	0.323	**< 0.001**	0.407	0.616	0.565	0.514
Light (12 h)	22.131 (1.884)^*^	21.715 (0.882)^*^	22.981 (1.567)^*^	23.934 (1.368)^*^	20.008 (1.421)^*^	19.651 (1.523)^**^	20.058 (1.068)	21.322 (1.559)	0.755	0.322	**0.002**	0.799	0.355	0.043	0.862
**CO**_2_ **production (ml/min/kg)**															
**Pre**															
24 h	24.117 (1.697)	23.990 (1.735)	24.387 (2.009)	25.973 (2.020)	21.032 (1.070)	21.640 (1.453)	21.278 (1.173)	20.891 (0.810)	**< 0.001**						
Dark (12 h)	27.470 (2.472)	27.095 (3.166)	27.393 (2.336)	29.644 (2.922)	23.732 (2.025)	25.090 (2.352)	24.965 (1.540)	24.246 (1.640)	**< 0.001**						
Light (12 h)	20.770 (1.052)	20.888 (1.155)	21.384 (2.485)	22.307 (1.525)	18.362 (0.894)	18.190 (1.251)	17.608 (1.083)	17.560 (0.535)	**< 0.001**						
**Post**															
24 h	23.667 (1.878)	23.226 (0.797)	24.390 (1.644)	25.836 (1.635)	20.584 (1.270)	20.821 (1.283)	21.880 (1.695)	23.223 (1.201)^**^	0.167	*0.083*	**< 0.001**	0.695	0.804	**0.045**	0.595
Dark (12 h)	27.506 (2.122)	26.890 (1.466)	28.475 (1.490)	29.980 (2.559)	22.946 (1.295)	23.926 (1.716)	26.010 (2.733)	27.077 (2.191)^*^	0.048	0.154	**< 0.001**	0.571	0.294	0.282	0.322
Light (12 h)	19.839 (2.035)	19.567 (1.628)	20.313 (2.397)	21.692 (1.404)	18.225 (1.937)	17.717 (1.481)	17.751 (1.110)	19.369 (1.655)^*^	0.992	0.207	**0.034**	1	0.417	**0.034**	0.785
**RQ (CO**_2_ **out/O**_2_ **in)**															
**Pre**															
24 h	0.863 (0.031)	0.865 (0.050)	0.883 (0.035)	0.894 (0.035)	0.898 (0.035)	0.896 (0.038)	0.876 (0.026)	0.899 (0.018)	0.075						
Dark (12 h)	0.882 (0.046)	0.882 (0.083)	0.913 (0.058)	0.932 (0.034)	0.938 (0.034)	0.934 (0.037)	0.914 (0.033)	0.944 (0.026)	**0.012**						
Light (12 h)	0.844 (0.043	0.848 (0.032)	0.853 (0.046)	0.857 (0.046)	0.859 (0.043)	0.858 (0.044)	0.837 (0.027)	0.854 (0.024)	0.901						
**Post**															
24 h	0.919 (0.028)^*^	0.912 (0.049)	0.887 (0.053)	0.924 (0.050)	0.928 (0.058)	0.918 (0.056)	0.904 (0.035)	0.931 (0.034)^*^	0.986	0.322	0.518	0.787	0.839	0.097	0.894
Dark (12 h)	0.947 (0.039)^*^	0.927 (0.056)	0.894 (0.064)	0.941 (0.054)	0.950 (0.056)	0.936 (0.056)	0.928 (0.049)	0.957 (0.038)	0.663	0.423	0.427	0.822	0.466	**0.038**	0.654
Light (12 h)	0.890 (0.025)	0.897 (0.045)	0.881 (0.064)	0.907 (0.055)	0.905 (0.065)^*^	0.900 (0.062)	0.881 (0.027)^**^	0.905 (0.045)^*^	0.68	0.319	0.704	0.791	0.712	0.357	0.832
**Energy expenditure (kcal/h)**															
**Pre**															
24 h	1.616 (0.169)	1.602 (0.152)	1.565 (0.097)	1.666 (0.155)	1.669 (0.150)	1.691 (0.145)	1.738 (0.173)	1.660 (0.141)	**0.04**						
Dark (12 h)	1.809 (0.177)	1.782 (0.149)	1.717 (0.124)	1.844 (0.155)	1.827 (0.202)	1.908 (0.230)	1.974 (0.216)	1.858 (0.153)	**0.027**						
Light (12 h)	1.422 (0.165)	1.422 (0.175)	1.413 (0.114)	1.488 (0.167)	1.513 (0.119)	1.474 (0.092)	1.504 (0.136)	1.463 (0.148)	0.133						
**Post**															
24 h	1.711 (0.156)^*^	1.737 (0.132)^*^	1.750 (0.146)^*^	1.930 (0.234)^*^	1.682 (0.113)	1.711 (0.110)	1.857 (0.213)^**^	1.893 (0.203)^*^	0.466	0.116	**0.001**	0.415	0.462	0.35	0.398
Dark (12 h)	1.950 (0.161)^*^	1.993 (0.197)^*^	2.035 (0.183)^*^	2.210 (0.274)^*^	1.846 (0.162)	1.943 (0.164)	2.175 (0.319)^*^	2.175 (0.311)^**^	0.288	0.188	**< 0.001**	0.607	0.276	0.881	0.334
Light (12 h)	1.472 (0.171)	1.482 (0.123)	1.465 (0.132)	1.649 (0.214)^*^	1.517 (0.116)	1.479 (0.089)	1.538 (0.121)	1.612 (0.126)^*^	0.985	0.104	**0.027**	0.262	0.963	**0.044**	0.665
**Activity index (AU)**															
**Pre**															
24 h	0.052 (0.020)	0.059 (0.026)	0.048 (0.012)	0.048 (0.014)	0.029 (0.020)	0.042 (0.016)	0.039 (0.016)	0.039 (0.013)	**0.005**						
Dark (12 h)	0.078 (0.034)	0.085 (0.039)	0.062 (0.015)	0.066 (0.019)	0.052 (0.030)	0.060 (0.025)	0.058 (0.025)	0.056 (0.019)	**0.016**						
Light (12 h)	0.027 (0.008)	0.033 (0.020)	0.035 (0.013)	0.031 (0.013)	0.026 (0.012)	0.024 (0.014)	0.021 (0.008)	0.022 (0.008)	**0.007**						
**Post**															
24 h	0.061 (0.021)	0.052 (0.018)	0.057 (0.022)	0.057 (0.015)	0.038 (0.019)	0.034 (0.009)	0.045 (0.019)	0.042 (0.012)	0.286	0.381	0.404	0.921	0.419	0.542	0.654
Dark (12 h)	0.086 (0.034)	0.074 (0.030)	0.085 (0.029)	0.083 (0.028)	0.050 (0.027)	0.049 (0.015)	0.069 (0.032)	0.059 (0.020)	0.204	0.36	0.172	0.935	0.431	0.96	0.472
Light (12 h)	0.036 (0.012)	0.031 (0.010)	0.028 (0.015)	0.031 (0.007)	0.026 (0.013)	0.019 (0.005)	0.020 (0.006)	0.025 (0.010)	0.842	0.611	0.415	0.909	0.538	0.065	0.694
**Feed consumption (g/24 h)**															
Pre	9.571 (2.760)	10.857 (4.706)	11.429 (2.507)	13.143 (3.934)	12.111 (2.088)	12.667 (3.708)	11.000 (2.179)	11.556 (1.944)	0.453						
Post	14.000 (2.082)^*^	14.429 (2.820)	12.857 (4.298)	17.000 (4.583)	15.000 (5.244)^*^	13.778 (3.420)	13.667 (4.848)	15.778 (6.016)	0.827	0.227	0.641	0.414	0.865	0.121	0.932
**Water intake (g/24 h)**															
Pre	4.143 (4.811)	6.571 (4.826)	10.429 (6.294)	6.286 (6.849)	4.333 (4.330)	7.889 (9.623)	2.222 (2.333)	8.000 (8.958)	0.459						
Post	16.000 (2.944)^*^	17.000 (2.449)^*^	15.000 (3.162)	17.714 (6.102)	9.222 (8.090)	18.333 (11.102)	11.222 (9.148)^*^	17.333 (14.248)^*^	0.303	**0.034**	0.935	0.191	0.883	0.883	0.59

^*^Stars show pre-post differences within a group analyzed via Wilcoxon signed ranks test.

Main effects examined via univariate analysis.

When examining the effect of line in POST measurements, BCAA-supplementation and running were used as covariates. Statistically significant findings marked in bold. ^*^p < 0.050, ^**^p < 0.010.

BCAA supplementation was associated with higher water intake, while voluntary running was associated with higher O_2_ consumption, CO_2_ production, and energy expenditure at all time periods (*p* ≤ 0.034, Table 2). BCAA supplementation and running were interactively associated with higher CO_2_ production (24 h and light) and RQ during the active hours (dark), as well as higher energy expenditure during light hours (*p* ≤ 0.045, Table 2).

Paired analysis (PRE vs. POST) showed that the O_2_ consumption decreased in all study HCR groups in at least one time period (24 h, dark or light, *p* ≤ 0.050, Table 2). Same was true for all the LCR groups except for LCR-CTRL-RUNNER, where no change was observed (Table 2). A significant change in CO_2_ production was observed only in LCR-BCAA-RUNNER group, where CO_2_ production was increased after the intervention (*p* ≤ 0.050, Table 2). An increase was observed in RQ (24 h and dark) in HCR-CTRL-NONRUNNER and during light time in LCR-CTRL-NONRUNNER groups, indicating a shift of using more carbohydrates for energy production (*p* ≤ 0.050, Table 2).

### 3.3 Voluntary running combined with BCAA supplementation was associated with larger heart and liver masses and smaller muscle-to-body-mass ratio in HCR rats

Tissue masses of heart, liver and hind limb skeletal muscles [soleus, extensor digitorum longus [EDL], plantaris, and gastrocnemius] are presented in Supplementary Table 1. Before including covariates in the multivariate model, rat line had a significant effect on body mass, plantaris, and gastrocnemius masses and heart- and liver-to-body-mass ratio (*p* < 0.001, *data not shown*). When including the covariates (BCAA supplementation and voluntary running), the effect of line was no longer significant (Supplementary Table 1). Line, BCAA supplementation and running were interactively associated with smaller muscle-to-body-mass ratio in HCRs and larger in LCRs (*p* = 0.015, Supplementary Table 1). Voluntary running combined with BCAA supplementation was associated with larger heart and liver masses and smaller muscle-to-body-mass ratio, whereas voluntary running alone was associated with larger heart- and liver-to-body-mass ratios in HCRs (*p* ≤ 0.050, Supplementary Table 1). In LCRs voluntary running was associated with larger heart and soleus masses as well as larger heart- and liver-to-body-mass ratios (*p* ≤ 0.050, Supplementary Table 1).

### 3.4 HCR and LCR rat lines can be distinguished by serum metabolome

From the collected serum we investigated the effects of intrinsic aerobic capacity, BCAA-supplementation, voluntary running, and the combination of BCAA-supplementation and voluntary running on metabolome.

**Figure 3:**
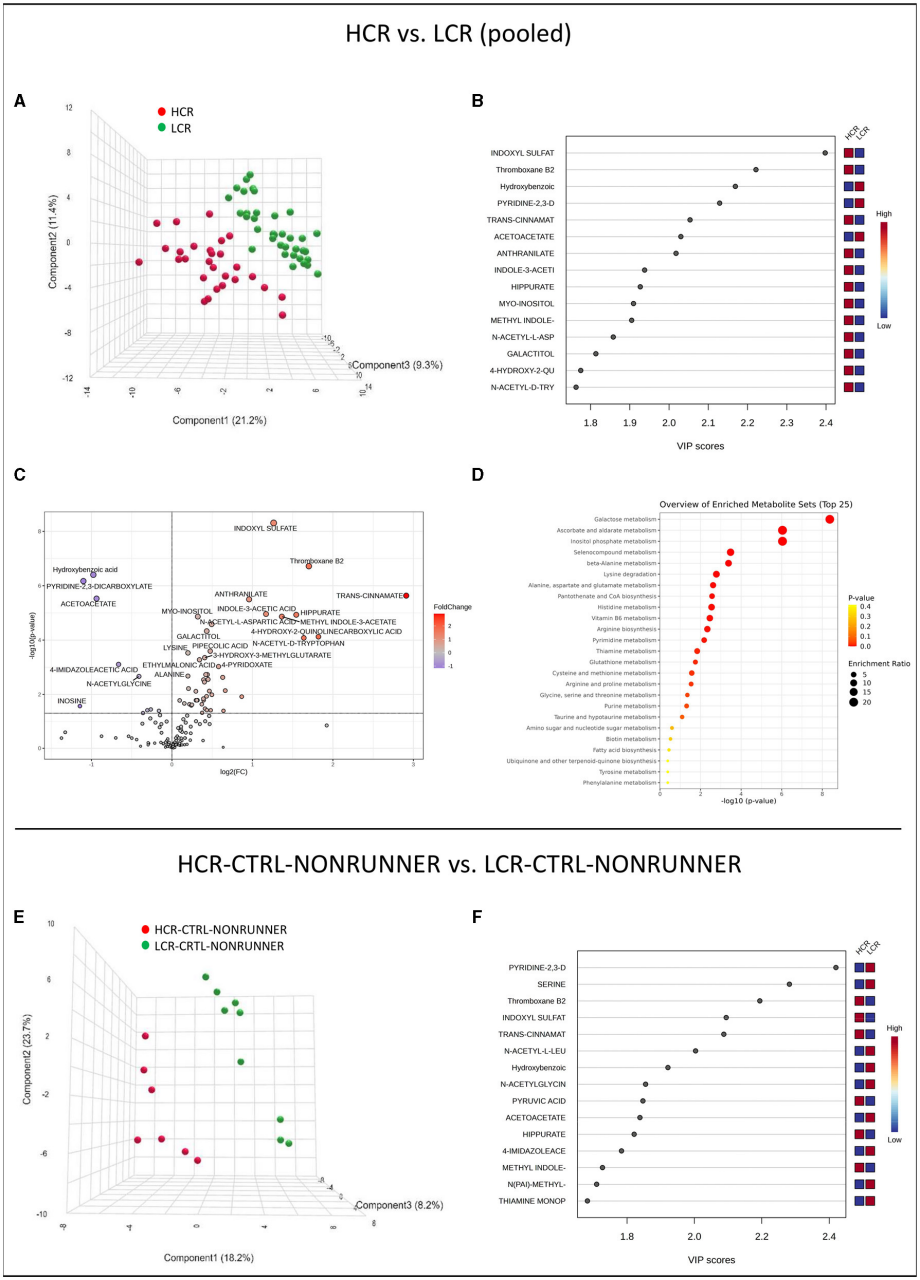
HCR and LCR rat lines can be distinguished by serum metabolome. PLS-DA 3D scores plot **(A)**, PLS-DA VIP scores **(B)**, volcano plot illustration of significantly changed metabolite relative abundancies **(C)**, and top 25 enriched metabolic pathways identified by MSEA **(D)** when comparing pooled HCR and LCR data. PLS-DA 3D scores plot **(E)** and PLS-DA VIP scores **(F)** when comparing HCR-CTRL-RUNNER to LCR-CTRL-RUNNER group. HCR, High-capacity runner; LCR, Low-capacity runner.

First, we analyzed the serum metabolomes of HCR and LCR lines, i.e., we pooled the data of all HCRs and all LCRs to examine metabolite differences between the rat lines. Partial least squares regression (PLS–DA) analysis showed separation of the rat lines into two different categories (Figure 3A). The separating metabolites in VIP plots were especially indoxyl sulfate and thromboxane B2 levels and lower hydroxybenzoic and pyridine-2,3-dicarboxylicate (quinolinic acid) levels, which were higher in HCR compared with LCR line (Figure 3B). Analysis of metabolite relative abundance illustrated in Volcano plot revealed in total 57 metabolite levels differed significantly in *t*-test (*p* ≤ 0.050, FC ≥ 1) between the rat lines, of which 48 were higher in HCR compared to LCR (*p* ≤ 0.049, Figure 3C, Supplementary Table 2). Furthermore, Metabolite Set Enrichment Analysis (MSEA) of the pooled HCR and LCR data revealed 14 metabolic pathways that were affected by the rat line (FDR-adjusted *p*-value ≤ 0.050, Figure 3D, Supplementary Table 3). Greatest differences were found in galactose, ascorbate and aldarate, and inositol phosphate metabolism, which were enriched in HCR compared with LCR line (Figure 3D, Supplementary Table 3). Heatmap clustering of the metabolites comparing pooled HCR and LCR lines is presented in Supplementary Figure 2.

When comparing the control groups (HCR-CTRL-NONRUNNER vs. LCR-CTRL-NONRUNNER), PLS-DA still showed separation between the rat lines (Figures 3E, F). However, neither analysis of metabolite relative abundance nor MSEA analysis showed significant differences between the two groups (*p* ≥ 0.195, Supplementary Tables 2, 3).

### 3.5 BCAA supplementation had more prominent effect on the serum metabolome of LCRs than HCRs

**Figure 4:**
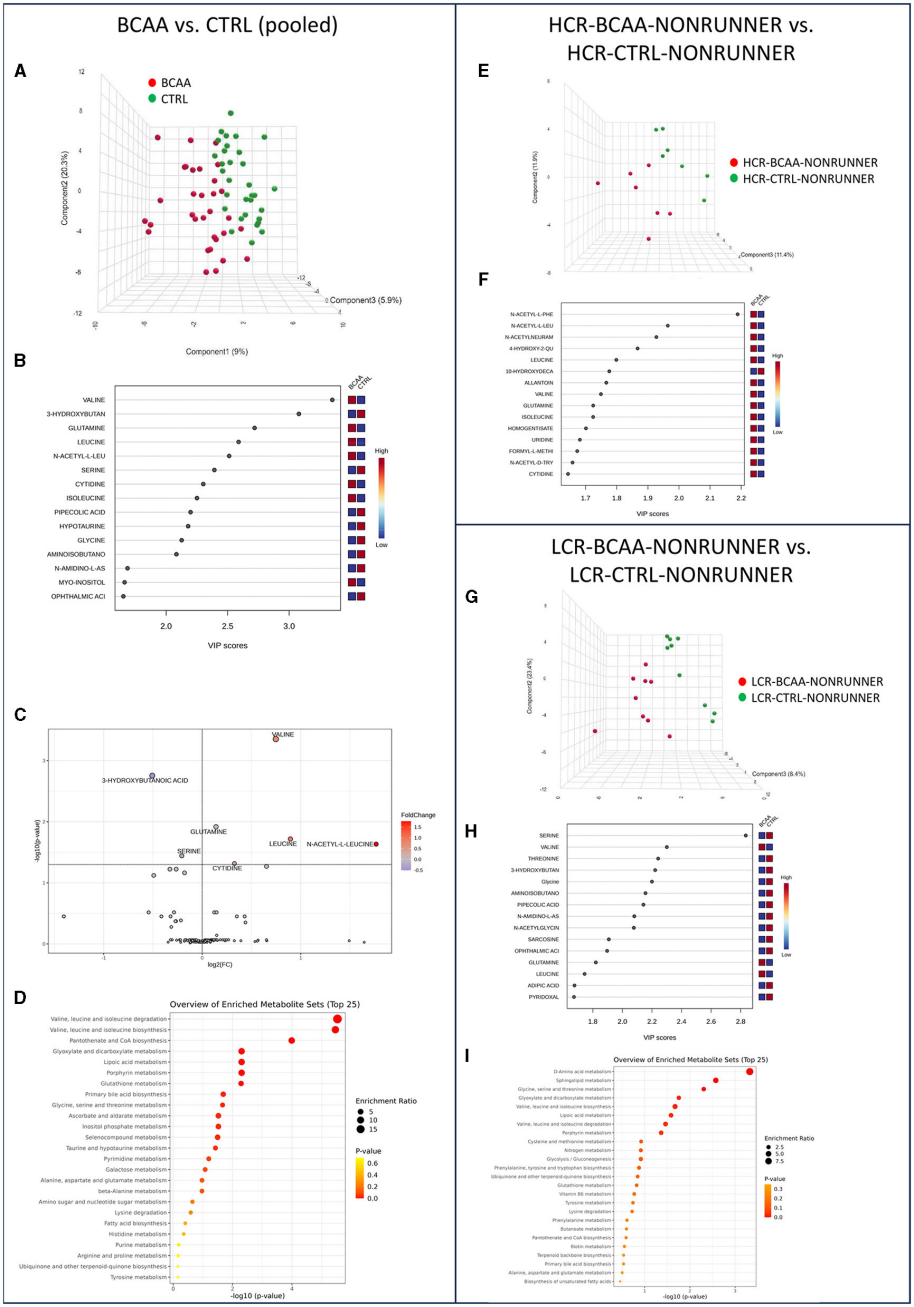
BCAA supplementation had more prominent effect on the serum metabolome of LCRs than HCRs. PLS-DA 3D scores plot **(A)**, PLS-DA VIP scores **(B)**, volcano plot illustration of significantly changed metabolite relative abundancies **(C)**, and top 25 enriched metabolic pathways identified by MSEA **(D)** when comparing pooled BCAA and control fed groups. PLS-DA 3D scores plot **(E)** and PLS-DA VIP scores **(F)** when comparing HCR-BCAA-NONRUNNER to HCR-CTRL-NONRUNNER group and PLS-DA 3D scores plot **(G)**, PLS-DA VIP scores **(H)**, and top 25 enriched metabolic pathways identified by MSEA **(I)** when comparing LCR-BCAA-NONRUNNER to LCR-CTRL-NONRUNNER group. HCR, High-capacity runner; LCR, Low-capacity runner.

Next, we studied the effects of BCAA supplementation on the serum metabolome of HCRs and LCRs. PLS–DA analysis showed again separation of the rat lines into two different categories, BCAA diet and control diet (Figure 4A). According to PLS-DA VIP scores, the rats in BCAA diet had higher valine, leucine, and isoleucine level than the control fed rats, as expected (Figure 4B). Analysis of metabolite relative abundance illustrated in Volcano plot revealed in total seven metabolite levels that were significantly different between the groups, of which five (valine, glutamine, leucine, acetyl leucine, and cytidine) were higher and two (3-hydroxybutanoic acid and serine) lower in BCAA diet compared to control diet groups (*p* ≤ 0.048, Figure 4C, Supplementary Table 4). MSEA showed seven metabolic pathways that were affected by the BCAA supplementation, with expectedly, the greatest differences found in BCAA metabolism (*p* ≤ 0.024, Figure 4D, Supplementary Table 5). Heatmap clustering of the metabolites comparing pooled BCAA and control diet groups is presented in Supplementary Figure 3.

In HCRs BCAA diet did not induce any significant effects on the serum metabolite level or in metabolic pathways (MSEA; *p* ≥ 0.524, Figures 4E, F, Supplementary Tables 4, 5). In LCRs the changes were more prominent than in HCRs. PLS-DA separated rats on BCAA and control diets showing again higher valine and leucine levels in the BCAA fed rats (Figures 4G, H). MSEA showed a significant enrichment of amino acid metabolism in the LCRs on BCAA diet (*p* = 0.024, Figure 4I, Supplementary Table 5). More detailed examination at the amino acid pathway showed that BCAA fed LCRs had higher serine level than control fed LCRs in serum (*p* = 0.006, Supplementary Table 8).

### 3.6 Voluntary running alone had minor effects on serum metabolome

**Figure 5:**
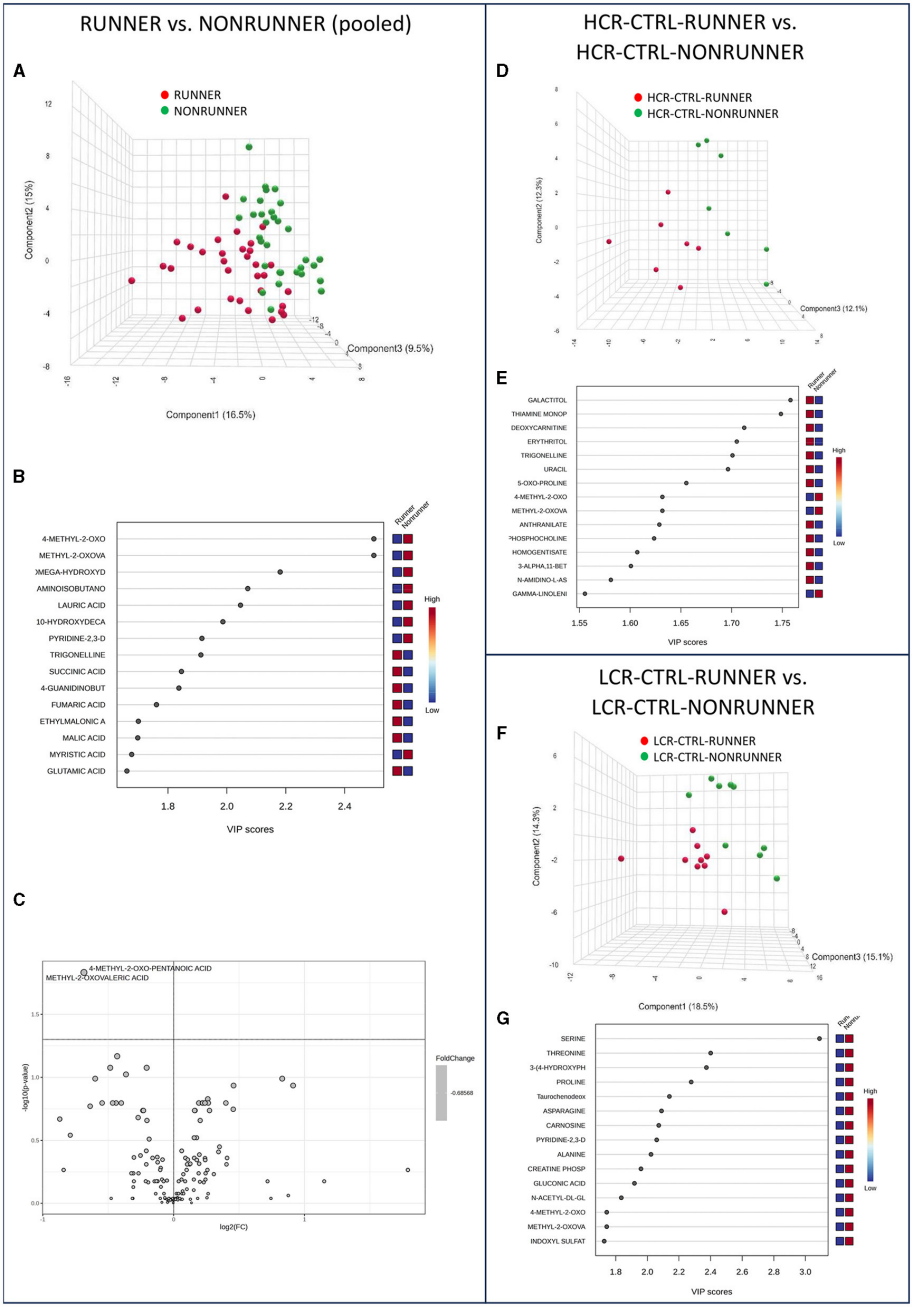
Voluntary running alone had minor effects on serum metabolome. PLS-DA 3D scores plot **(A)** and PLS-DA VIP scores **(B)**, and volcano plot illustration of significantly changed metabolite relative abundancies **(C)**, when comparing pooled data of all HCRs and LCRs with voluntary running (RUNNER) vs. controls (NONRUNNER). PLS-DA 3D scores plot **(D)** and PLS-DA VIP scores **(E)** when comparing HCR-CTRL-RUNNER to HCR-CTRL-NONRUNNER and PLS-DA 3D scores plot **(F)** and PLS-DA VIP scores **(G)** when comparing LCR-CTRL-RUNNER to LCR-CTRL-NONRUNNER. HCR, High-capacity runner; LCR, Low-capacity runner.

To continue our analysis, we examined the effects of voluntary running to the metabolome of HCRs and LCRs. In the pooled data (RUNNERs vs. NONRUNNERs) PLS-DA could separate the rats into two categories, especially based on 4-methyl-2-oxo-pentanoic acid and metyl-2-oxovaleric acid levels (Figures 5A, B). The aforementioned metabolites were also significantly higher in the analysis of metabolite relative abundance of pooled runner vs. non-runner groups (*p* = 0.015, Figure 5C, Supplementary Table 6). MSEA revealed no significant pathway enrichment differences between the control voluntary running animals, yet BCAA degradation was close of being significant (*p* = 0.062, Supplementary Table 7). When examining HCRs and LCRs separately, voluntary running did not induce any significant effects on the serum metabolite levels or metabolite pathways analyzed via MSEA in either of the rat lines (*p* ≥ 0.154, Figures 5D–G, Supplementary Tables 6, 7).

### 3.7 The combined effect of BCAA supplementation and voluntary running on serum metabolome was more prominent than either treatment alone

**Figure 6:**
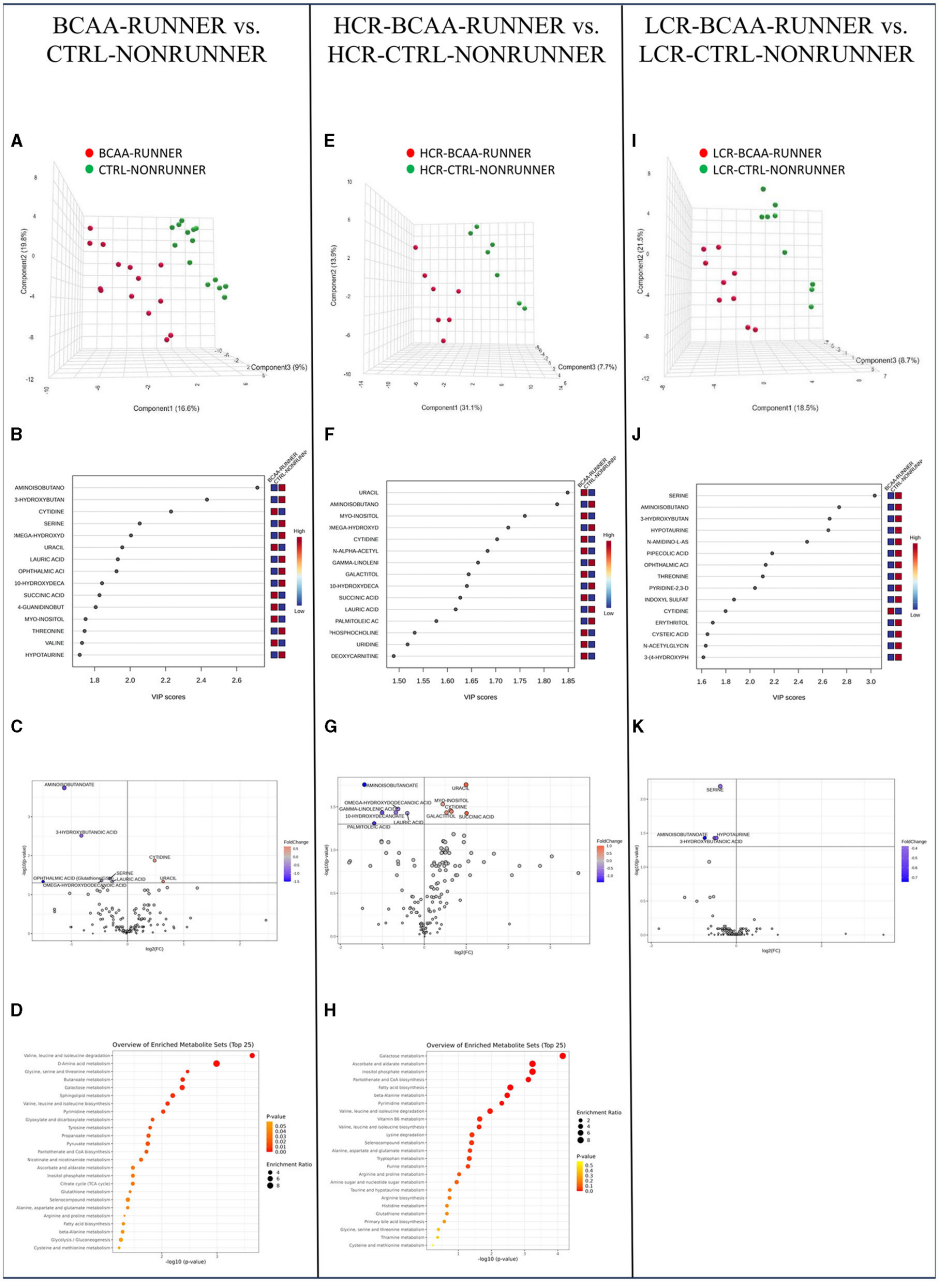
The combined effect of BCAA supplementation and voluntary running on serum metabolome was more prominent than either treatment alone. PLS-DA 3D scores plot **(A)**, PLS-DA VIP scores **(B)**, volcano plot illustration of significantly changed metabolite relative abundancies **(C)**, and top 25 enriched metabolic pathways identified by MSEA **(D)** when comparing pooled HCR and LCR with treatments (BCAA-RUNNER) with controls (CTRL-NONRUNNER). PLS-DA 3D scores plot **(E)**, PLS-DA VIP scores **(F)**, volcano plot illustration of significantly changed metabolite relative abundancies **(G)**, and top 25 enriched metabolic pathways identified by MSEA **(H)** when comparing HCR-BCAA-RUNNER with HCR-CTRL-NONRUNNER. PLS-DA 3D scores plot **(I)**, PLS-DA VIP scores **(J)**, and volcano plot illustration of significantly changed metabolite relative abundancies **(K)** when comparing LCR-BCAA-RUNNER with LCR-CTRL-NONRUNNER. HCR, High-capacity runner; LCR, Low-capacity runner.

Our final analysis showed that combined BCAA-supplementation and voluntary running had clear effects on serum metabolome compared to BCAA-supplementation or running exercise alone. In pooled HCR and LCR data, PLS-DA separated clearly the treated and control animals, with the greatest difference being in aminoisobutanoate, 3-hydroxybutyric acid, cytidine and serine levels (Figures 6A, B). Analysis of metabolite relative abundance illustrated in Volcano plot revealed in total eight significantly changed metabolites, with two metabolite (cytidine and uracil) levels being higher and five (aminoisobutanoate, 3-hydroxybutanoic acid, serine, omega-hydroxydodecanoic acid, ophthalmic acid and lauric acid) being lower in the BCAA and running treated groups compared to untreated groups (*p* ≤ 0.046, Figure 6C, Supplementary Table 8). MSEA showed enrichment of five significantly changed metabolite pathways, including BCAA degradation, amino acid, glycine, serine and threonine, and butanoate and galactose metabolism (*p* ≤ 0.043, Figure 6D, Supplementary Table 9).

In HCRs PLS-DA separated treated vs. controls into two groups, with the greatest differences being in aminoisobutanoate and uracil levels (Figures 6E, F). We observed 12 metabolite levels, including higher uracil, myo-inositol, and cytidine levels and lower aminoisobutanoate and omega-hydroxydodecanoic acid level in the treated vs. control group in the HCR line (*p* ≤ 0.033, Figure 6G, Supplementary Table 8). Via MSEA analysis we found eight significantly enriched metabolic pathways including galactose metabolism, phosphatidylinositol signaling, pantothenate and CoA biosynthesis and metabolism of ketone bodies (*p* ≤ 0.046, Figure 6H, Supplementary Table 9).

In LCRs, PLS-DA was separated the two groups based on especially serine and aminoisobutanoate levels (Figures 6I, J). Analysis of metabolite relative abundance illustrated in Volcano plot revealed four significantly lower metabolite levels (serine, aminoisobutanoate, 3-hydroxybutanoic acid, and hypotaurine) when comparing the treated groups with controls within LCR line (*p* ≤ 0.037, Figure 6K, Supplementary Table 8). However, MSEA revealed no significantly enriched metabolic pathways (*p* ≥ 0.168, Supplementary Table 9).

## 4 Discussion

This research explored the impact of dietary BCAA supplementation and voluntary running on metabolism in rat lines with distinct difference in intrinsic running capacity. Our findings confirmed the successful administration of BCAA (2x) in our study design. In LCR rats, BCAA supplementation led to a decrease in daily voluntary running distance. Notably, we are the first to demonstrate that BCAA supplementation has a more pronounced effect on the serum metabolome of LCRs compared to HCRs, showing enrichment of serine metabolism. Additionally, while voluntary running had minimal impact on serum metabolome in both HCRs and LCRs, our results show that the combined effects of BCAA supplementation and voluntary running were more significant than either treatment alone.

### 4.1 Intrinsic aerobic capacity affects serum metabolome

HCR and LCR rat lines exhibit differences not only in their maximal running capacity but also in body mass and several indicators of metabolic health (24, 28, 29, 47). In the present study HCRs exhibited lower body mass, and higher voluntary running activity and maximal running capacity than LCRs. We also observed distinct variations in the serum metabolome between the HCR and LCR rat lines, consistent with previous findings (33, 48). Specifically, HCRs had higher levels of indoxyl sulfate and thromboxane B2 compared to LCRs. Indoxyl sulfate, a uremic toxin derived from tryptophan metabolism (49), and thromboxane B2, a platelet-derived metabolite (50), were both elevated in HCRs. Interestingly, despite the better overall health observed in HCRs, higher levels of these metabolites are associated with cardiovascular complications (51, 52). Further studies are needed to fully understand the implications of these results.

Previous observations have highlighted the difference in lipid metabolism between the HCR and LCR rat lines (22, 32). A recent study suggested that especially hub genes Acad1, Cd2bp2, Plb1, and Pla2g7 may share a role in the difference in aerobic capacity between HCR and LCR rat lines (53). While the possible role of Cd2bp2, Plb1, and Pla2g7 in BCAA metabolism remains uncovered, Acad1 likely influences the balance between BCAA utilization and fatty acid oxidation (53). As lipid and BCAA metabolism are interconnected (3), same genes may influence also BCAA utilization during endurance exercise.

According to MSEA analysis performed here, the HCR and LCR rat lines exhibit differences particularly in the metabolism of carbohydrates, amino acids and their derivatives. In our previous targeted metabolomics study, we observed significant changes in only three metabolites within the serum metabolome: HCRs had higher levels of isovalerylcarnitine, inosine, and hexanoylcarnitine compared with LCRs (33). Another study reported a total of 33 significantly changed metabolites in serum of HCR/LCR rats, with most belonging to amino acid metabolism (48). Additionally, Falegan et al. (54) found that HCR and LCR rat lines differ in alanine, aspartate, and glutamate levels in plasma. The observed differences between these studies can be attributed to differences in the rat generations used, the sex of the rats, as well as the age of the rats at euthanasia. In addition, differences in chosen metabolomics analytical techniques affect the obtained results. Of the aforementioned studies, Falegan et al. (54) utilized male rats of 13 months of age from generations 17–19, while our previous study was done in female rats of 9 months of age from the generations 23–27 of selection (33, 54). Closest to our current design is the study by Aon et al. (48), who utilized female rats of 6 months of age from the generations 35–38 of selection, who also detected several differing metabolites in serum of HCR and LCR lines. Despite the observed differences between the studies, many of the 57 significantly changed metabolite levels in our data were either amino acids or amino acid derivatives, similar to previous observations. The heatmap cluster analysis of the present study showed lower levels of hydrobenzoic acid and acetoacetate in the LCR line, which may indicate dysfunction related to amino acid metabolism or liver function (55). In summary, our present results support of the notion of the differences especially in the amino acid metabolism between the rat lines.

### 4.2 Voluntary running increased food intake and energy expenditure, but had only minor effects on serum metabolome

Thisstudy showed that voluntary running increased food intake and energy expenditure in both rat lines. To our surprise in LCR line the maximal running capacity increased in all the studied groups. This observation may be due to the improved running capacity induced by the maximal running tests that were repeated three times before and after the intervention. Since LCRs typically have low spontaneous activity level (28), the maximal running tests may improve their performance. However, the increase in the maximal running capacity was largest in the RUNNER groups.

The levels of only two metabolites were significantly lower in the RUNNER groups compared with NONRUNNERS; 4-methyl-2-oxo-pentanoic acid and methyl-2-oxovaleric acid. 4-methyl-2-oxo-pentanoic acid is metabolite involved in BCAA metabolism. Methyl-2-oxovaleric acid in turn is metabolite derived from the breakdown of leucine (56). Low levels of these metabolites may indicate issues with BCAA metabolism or impaired leucine catabolism. On the other hand, high 4-methyl-2-oxo-pentanoic level is associated with defects in BCAA metabolism (57). Our results indicate that voluntary running affects also the BCAA metabolism in HCR/LCR rat lines.

### 4.3 BCAA supplementation had more prominent effects on LCRs than HCRs

The results showed that BCAA supplementation had no effect on metabolism, spontaneous activity or tissue masses in either of the studied rat lines. However, BCAA supplementation decreased daily voluntary running distance in LCRs. We previously noticed similar effect when LCRs received whey protein (37). Voluntary running stayed low for 10 weeks during the whey supplementation (37). It seems that high BCAA level decreases voluntary running in LCRs but not in HCRs. Further studies are required to clarify this phenomenon.

When examining the serum metabolome of pooled HCR and LCR data, analysis revealed higher valine and leucine levels in the BCAA diet compared with control diet, as expected due to the BCAA supplementation. The Metabolic pathway enrichment and heatmap cluster analysis further revealed that also isoleucine level was elevated in the BCAA diet groups. Interestingly BCAA diet did not induce any significant effects on the serum metabolite level or in metabolic pathways in HCRs. In LCRs there was a significant enrichment of amino acid metabolism on BCAA diet, and serine was found to be the significantly differing amino acid increasing with BCAA supplementation. In mammals, serine biosynthesis occurs primarily through the phosphorylated pathway starting from 3-phosphoglycerate. Increased serum phosphocholine and glutathione levels are associated with BCAA supplementation (7). These changes may indirectly impact serine metabolism, yet the specific mechanisms remain uncovered. Low serine levels have been observed in patients with diabetes (58, 59) and pre-diabetic mice (60), while dietary supplementation with serine ameliorates diabetes (61). Thus, a low serine level may be significant factor in both diabetic and prediabetic conditions, though the potential mechanisms are not yet known. Accordingly, our results indicate that BCAA supplementation may change the metabolism of LCRs into healthier direction.

### 4.4 The combination of BCAA supplementation and voluntary running had distinct effects in HCR and LCR rat lines

The results show that combining BCAA supplementation with voluntary running had noticeably stronger effects than BCAAs or voluntary running alone. BCAA and running were interactively associated with higher RQ during the dark hours and higher energy expenditure during light hours. BCAA supplementation and running were interactively associated with smaller muscle-to-body-mass ratio in HCRs and larger in LCRs. In LCRs this was due to tendency of having slightly lower body mass and larger muscle masses (soleus, EDL, and plantaris) of the LCR-BCAA-RUNNER group compared with LCR-CTRL-NONRUNNER group. In HCRs the muscle masses of soleus, EDL, and plantaris also tended to be larger in HCR-BCAA-NONRUNNER compared with HCR-CTRL-RUNNER group, yet the body mass was also larger, mitigating the change in the muscle-to-body-mass ratio. As HCR rats have shown to have higher expression of genes related to oxidative phosphorylation and fatty acid and BCAA metabolisms in the skeletal muscle (22, 32), it is likely that the increase in muscle-to-body-mass -ratio of LCRs is not sufficient to compensate for the lower gene expression on energy metabolism related genes.

Combined BCAA diet and voluntary running had greater effects in the serum metabolome of HCRs than LCRs. In HCRs we observed 12 metabolite levels and eight metabolic pathways that were significantly changed, while in LCRs, only four metabolite levels were significantly changed with no enriched metabolic pathways. In all studied comparisons, we observed low levels of BCAA metabolite aminoisobutanoate and fatty acids (omega-hydroxydodecanoic acid, lauric acid, and/or 3-hydroxybutanoic acid). Growing body of literature suggest that BCAA metabolism, fatty acid metabolism and physical activity are interconnected (20, 21). In support, our present findings indicate that BCAA diet combined with voluntary running affect both BCAA and fatty acid metabolism.

Of the enriched metabolic pathways, the pooled data and HCRs had in common valine, leucine, and isoleucine degradation which again affirms successful supplementation of BCAAs. Excitingly, in HCRs also fatty acid biosynthesis pathway was significantly enriched. In our previous work, we proposed a hypothesis linking BCAA degradation to the intricate processes of lipid oxidation and storage within skeletal muscle (3). Briefly, our hypothesis proposes that transamination of BCAAs is critical for cytosolic oxaloacetate formation. It is metabolized to phosphoenolpyruvate for glyceroneogenesis, that is required in skeletal muscles for the storage of fatty acids as lipid droplets (3). We suggest that the intramyocellular lipid droplets are then utilized to provide energy both during extended exercise sessions and at rest (3). In agreement, we have previously shown, that BCAA deprivation decreases lipid oxidation and lipogenesis in myotubes (62). Our present results support the interconnection of BCAA and fatty acid metabolism in contributing to better metabolic health.

The greater effects of BCAA diet and voluntary running observed in HCRs is likely explained by two factors: higher relative BCAA intake and more active voluntary running compared to LCRs. Even though the absolute BCAA intake (g/d) was similar between the rat lines, the BCAA intake g/body mass was higher in the HCR line due to their lower body mass. In addition, similarly as we have observed previously (28, 39), HCRs ran voluntarily on a running wheel more than LCRs. These factors are most probably underlying the greater additive effects of BCAA diet and running in the HCR line.

## 5 Conclusions

Our findings are consistent with earlier studies showing that serum metabolome profiles can differentiate between HCR and LCR rat lines. We are the first to show that BCAA supplementation had more prominent effects on LCR than HCR rats. In LCR rats, BCAA supplementation led to a decrease in daily voluntary running distance accompanied with enrichment of serine metabolism in the serum metabolome. Our data shows that while voluntary running increased food intake and energy expenditure, it had only minor effects on serum metabolome. Our results revealed that the combined effects of BCAA supplementation and voluntary running on serum metabolome were more significant than either treatment alone. Combined BCAA diet and voluntary running had greater effects in HCRs than in LCRs, which is likely explained by larger relative BCAA intake and higher voluntary running activity. This data suggests that BCAA diet alone or combined with exercise benefits both metabolically unfit and fit individuals. Our present results support the interconnection of BCAA and fatty acid metabolism in contributing to metabolic health.

## Data Availability

The data presented in the study has been deposited in the MetaboLights repository, accession number MTBLS11132.
