# Supplementary material for: Branched-chain amino acid supplementation and voluntary running have distinct effects on the serum metabolome of rats with high or low intrinsic aerobic capacity
Source: Front Nutr. 2024 Nov 19;11:1450386. doi: 10.3389/fnut.2024.1450386 (PMC11611553; doi:10.3389/fnut.2024.1450386)
Supplement: Supplementary file 1 [file Data_Sheet_1.pdf]

Supplementary Material

Supplementary Figures and Tables

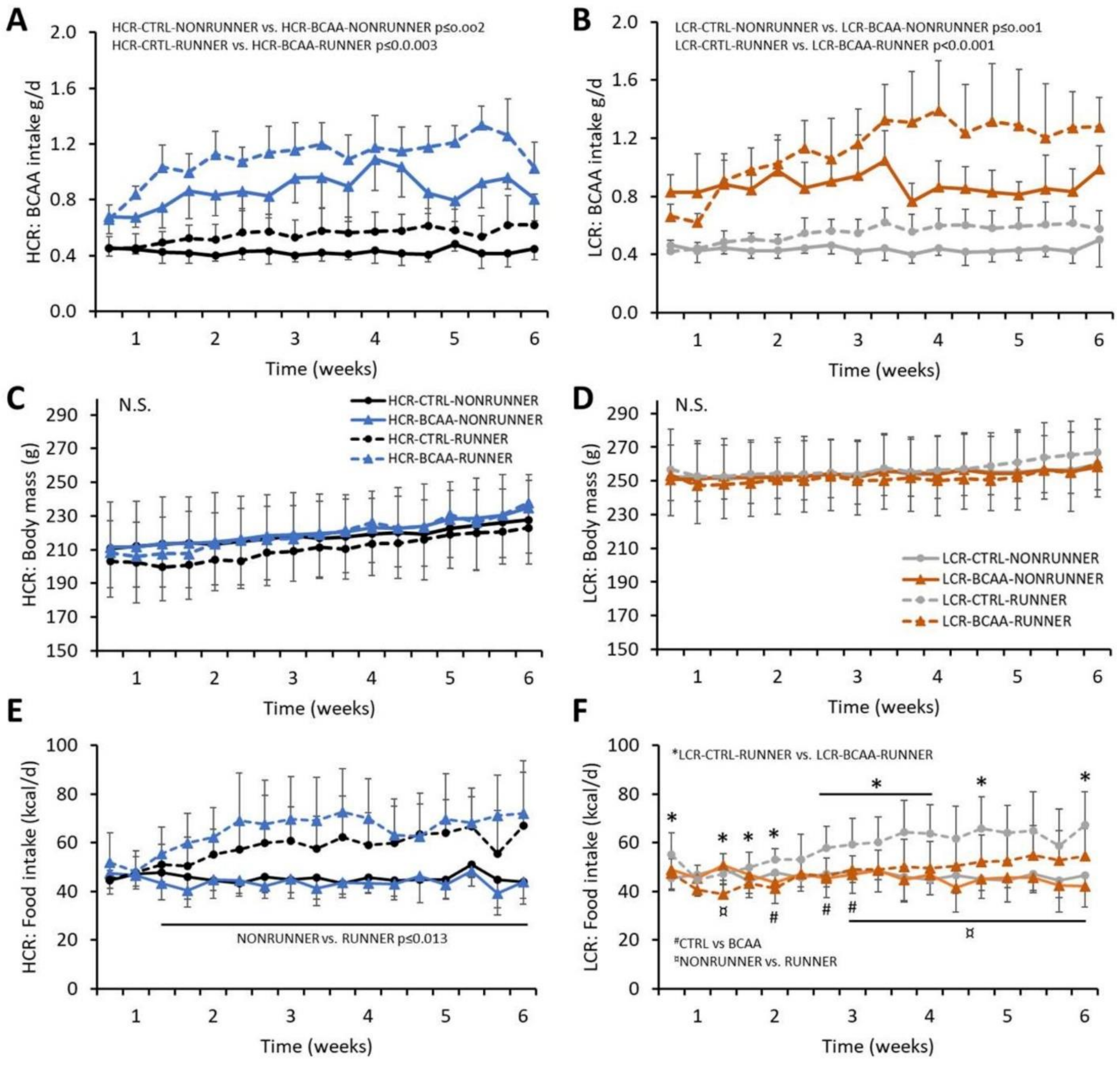

FIGURE S1. BCAA intake (**A-B**), body mass (**C-D**), and food intake (**E-F**) during the intervention in HCR and LCR rats. HCR=High-capacity runner, LCR=Low-capacity runner, CTRL=control diet, BCAA=Branched-chain amino acid diet. Data is presented as mean and SD. *p≤0.010.

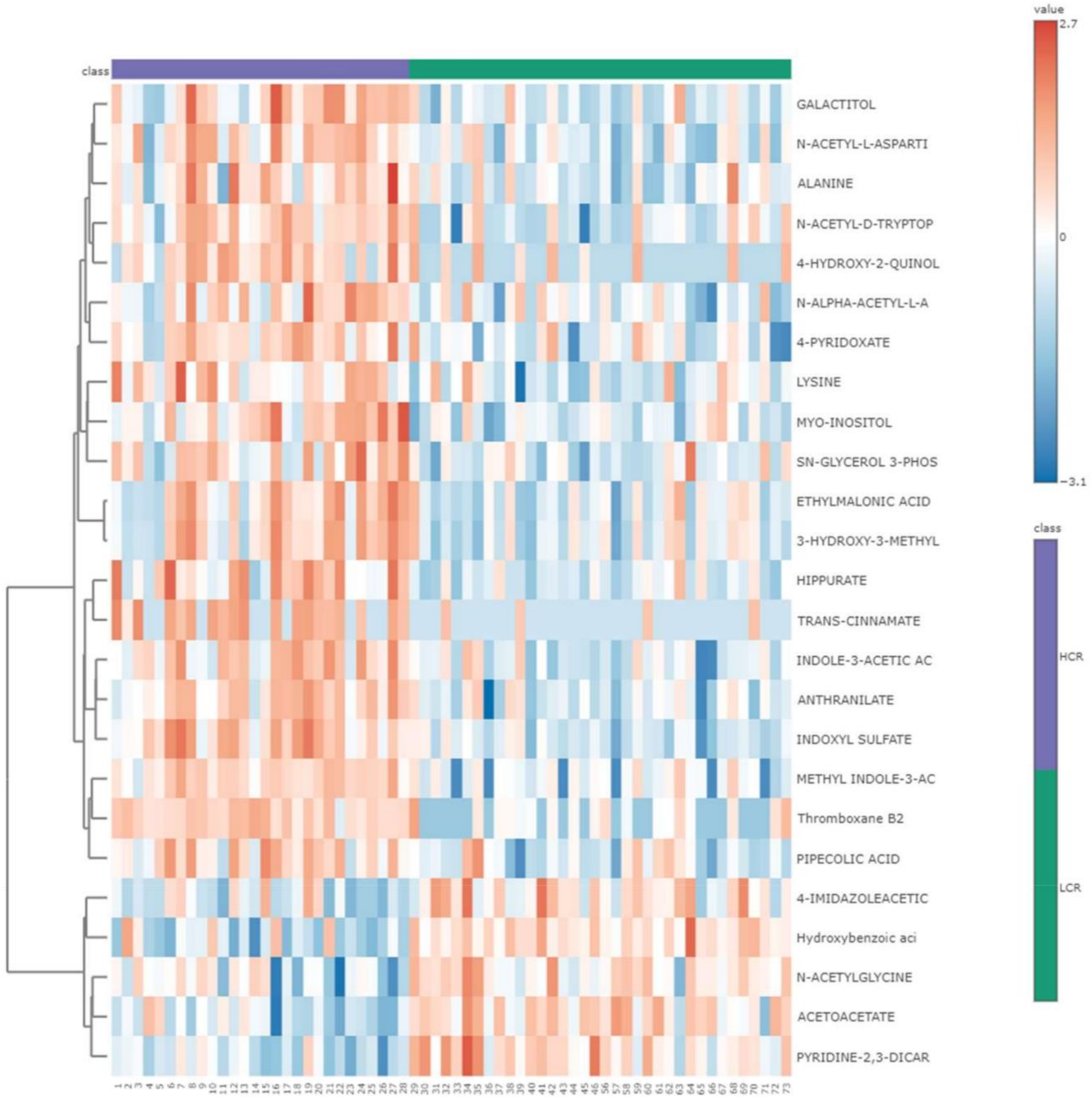

FIGURE S2. Heatmap clustering of the metabolites when comparing pooled HCR and LCR groups. HCR=High-capacity runner, LCR=Low-capacity runner.

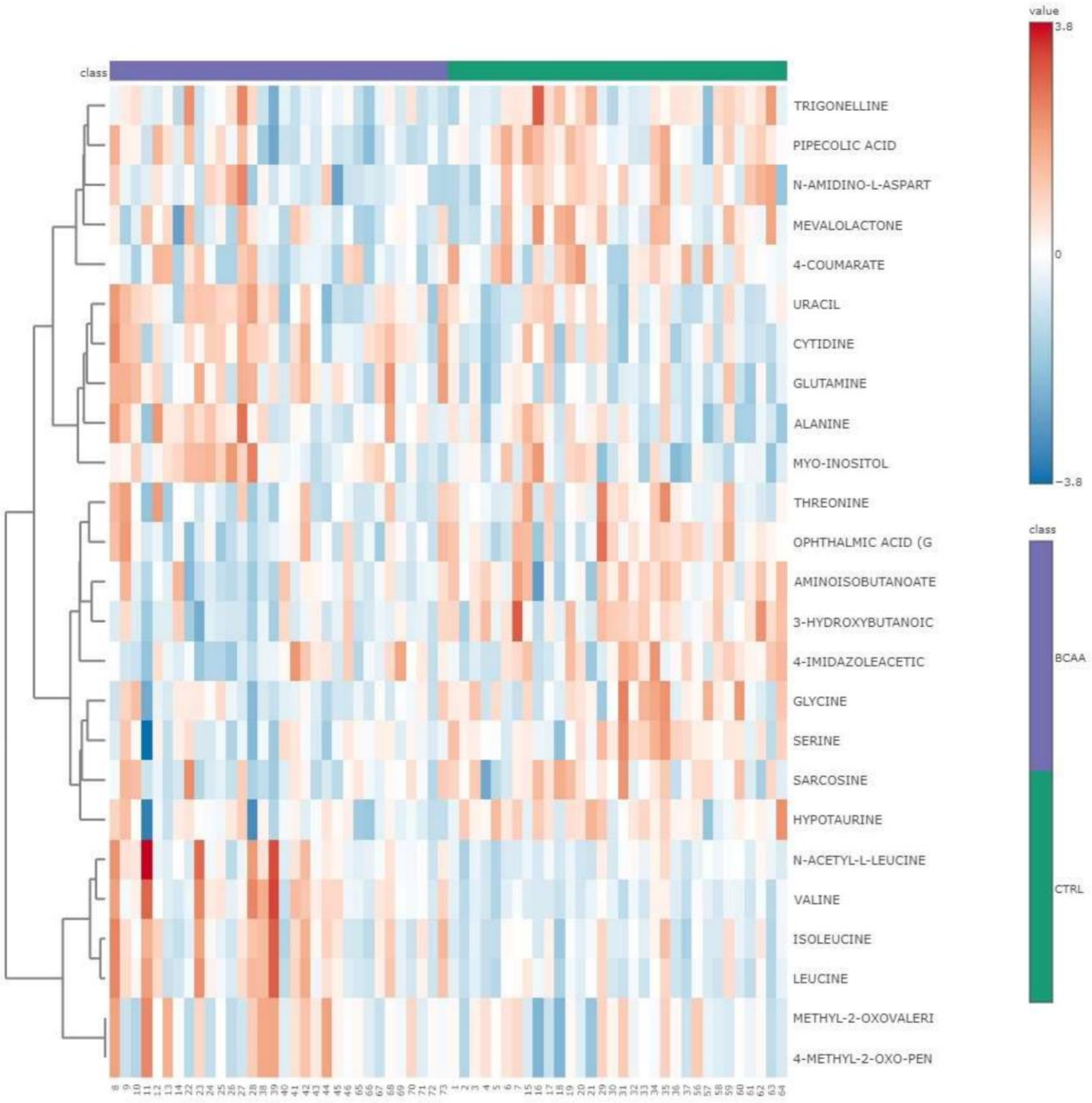

FIGURE S3. Heatmap clustering of the metabolites when comparing pooled BCAA and control diet groups. BCAA=Branched-chain amino acid, CTRL=control feed.

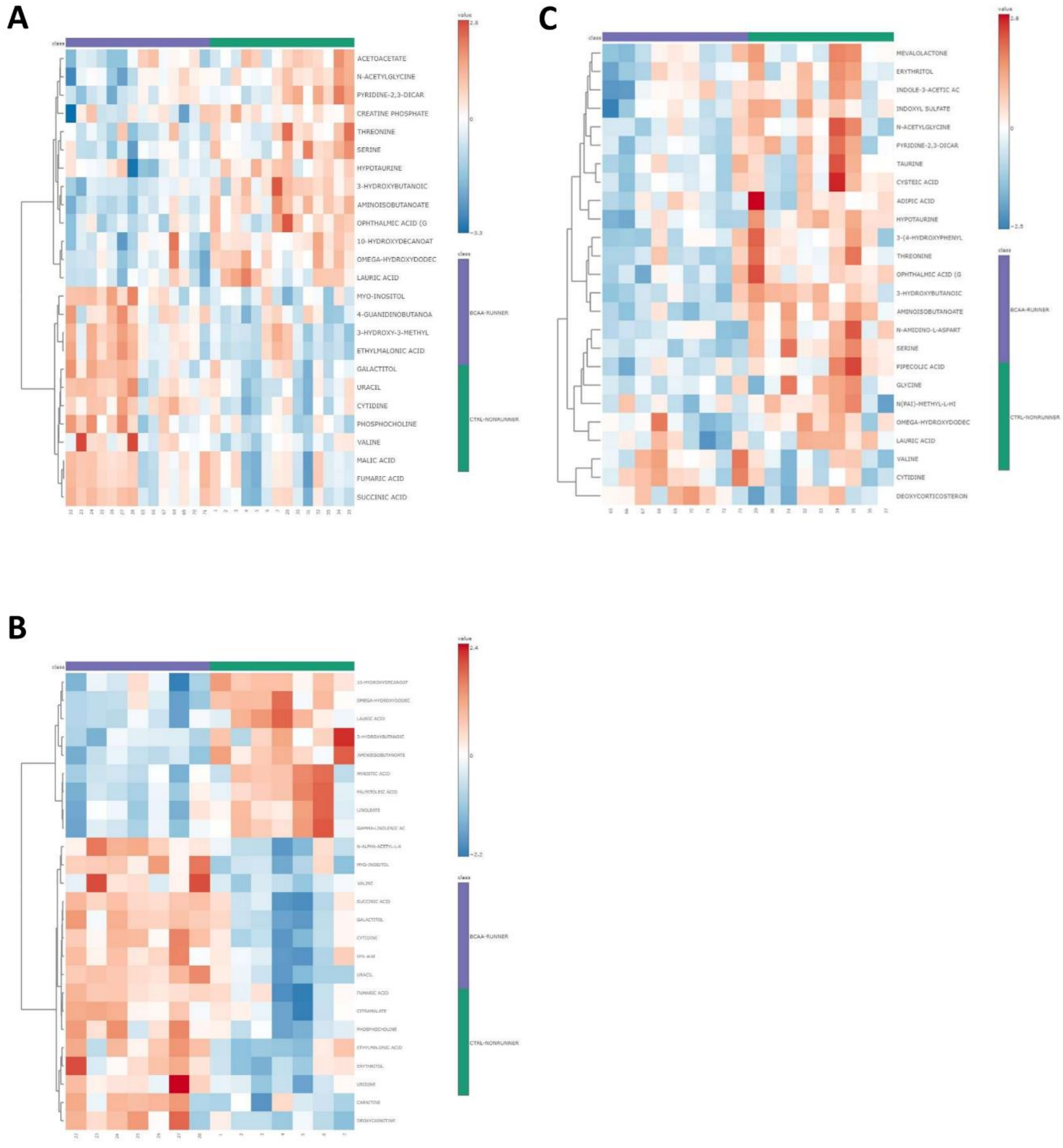

FIGURE S4. Heatmap clustering of the metabolites when comparing pooled treatment (BCAA-RUNNER) and control (CTRL- NONRUNNER) groups (**A**), HCR-BCAA-RUNNER vs. HCR-CTRL-NONRUNNER (**B**), and LCR-BCAA-RUNNER vs. LCR-CTRL-NONRUNNER (**C**). HCR=High-capacity runner, LCR=Low-capacity runner, BCAA=Branched-chain amino acid, CTRL=control feed.

Table S1. Tissue masses (g) and relative tissue masses (relative to body mass*100) in the study groups (mean±SD).

	**Group**								**p-values**					
	HCR-CTRL-NON-RUNNER	HCR-BCAA-NON-RUNNER	HCR-CTRL-RUNNER	HCR-BCAA-RUNNER	LCR-CTRL-NON-RUNNER	LCR-BCAA-NON-RUNNER	LCR-CTRL-RUNNER	LCR-BCAA-RUNNER	Line	BCAA	Running	Line* BCAA	Line* Running	BCAA* Running
Masses (g)														
Body mass	223 (26)	229 (14)	215 (19)	232 (28)	252 (20)	252 (13)	254 (19)	248 (20)	0.883	0.936	0.768	0.731	0.413	0.852
Heart	0.827 (0.078)	0.0879 (0.073)	0.924 (0.102)	1.059 (0.105)**	0.842 (0.108)	0.822 (0.052)	0.997 (0.158)#	0.933 (0.123)	0.711	0.978	0.210	0.741	0.275	0.726
Liver	6.542 (0.953)	6.536 (0.580)	7.393 (1.198)	8.331 (1.570)*	6.628 (0.856)	6.647 (0.383)	7.383 (0.947)	7.380 (0.778)	0.735	0.547	0.652	0.513	0.566	0.336
Soleus	0.097 (0.014)	0.104 (0.014)	0.107 (0.019)	0.111 (0.017)	0.098 (0.010)	0.100 (0.009)	0.119 (0.012)##	0.118 (0.011)##	0.880	0.462	*0.081*	0.817	0.619	0.653
EDL	0.123 (0.014)	0.127 (0.010)	0.118 (0.009)	0.126 (0.016)	0.125 (0.008)	0.124 (0.010)	0.132 (0.008)	0.127 (0.011)	0.575	0.864	0.929	0.823	0.226	0.986
Plantaris	0.250 (0.022)	0.249 (0.015)	0.243 (0.025)	0.253 (0.027)	0.270 (0.020)	0.271 (0.021)	0.277 (0.025)	0.275 (0.021)	0.897	0.817	0.834	0.698	0.458	0.717
Gastocnemius	1.225 (0.146)	1.242 (0.072)	1.147 (0.139)	1.097 (0.219)	1.388 (0.115)	1.343 (0.078)	1.360 (0.099)	1.385 (0.099)	0.321	0.870	0.582	0.318	0.658	0.977
Heart/body mass	0.372 (0.022)	0.384 (0.023)	0.430 (0.035)**	0.460 (0.036)***	0.334 (0.025)	0.327 (0.026)	0.393 (0.058)#	0.376 (0.036)#	0.575	0.870	*0.058*	0.861	0.588	0.815
Liver/body mass	2.927 (0.172)	2.852 (0.182)	3.428 (0.401)*	3.586 (0.381)***	2.628 (0.209)	2.635 (0.111)	2.904 (0.261)#	2.971 (0.132)###	0.595	0.947	0.219	0.515	0.895	0.239
Muscle/body mass	0.760 (0.026)	0.752 (0.025)	0.749 (0.032)	0.687 (0.081)*	0.748 (0.036)	0.728 (0.028)	0.744 (0.038)	0.769 (0.020	0.368	0.776	0.936	*0.084*	0.151	0.805

*compared to HCR-CTRL-NONRUNNER

#compared to LCR-CTRL-NONRUNNER

Muscle masses (soleus, extensor digitorum longus [EDL], plantaris and gastrocnemius) are presented as average of the right and left muscle.

Muscle mass to body mass ratio is presented as {[soleus (average, g)+ EDL (average, g) + plantaris (average, g) + gastrocnemius (average, g)]/body mass (g)}*100 to estimate the relative total hind limb muscle mass.

Table S2. Examining the effect of rat line on metabolite levels. All significantly changed metabolites of pooled HCR vs. LCR and twenty most significantly changed metabolites of HCR-CTRL-NONRUNNER vs. LCR-CTRL-NONRUNNER groups.

Comparison	Number	Metabolite	FC	log2(FC)	p-value (unadjusted)	FDR-adjusted p-value
HCR vs. LCR (pooled)	**1**	**Indoxyl Sulfate**	2.401	1.263	0.000	**0.000**
	**2**	**Thromboxane B2**	3.251	1.701	0.000	**0.000**
	**3**	**Hydroxybenzoic Acid**	0.508	-0.977	0.000	**0.000**
	**4**	**Pyridine-2,3-Dicarboxylate**	0.466	-1.101	0.000	**0.000**
	**5**	**Trans-Cinnamate**	7.520	2.911	0.000	**0.000**
	**6**	**Acetoacetate**	0.522	-0.937	0.000	**0.000**
	**7**	**Anthranilate**	1.943	0.958	0.000	**0.000**
	**8**	**Indole-3-Acetic Acid**	2.248	1.169	0.000	**0.000**
	**9**	**Hippurate**	2.917	1.544	0.000	**0.000**
	**10**	**Methyl Indole-3-Acetate**	2.576	1.365	0.000	**0.000**
	**11**	**Myo-Inositol**	1.249	0.321	0.000	**0.000**
	**12**	**N-Acetyl-L-Aspartic Acid**	1.407	0.493	0.000	**0.000**
	**13**	**Galactitol**	1.349	0.432	0.000	**0.000**
	**14**	**4-Hydroxy-2-Quinolinecarboxylic Acid**	3.530	1.820	0.000	**0.000**
	**15**	**N-Acetyl-D-Tryptophan**	3.113	1.638	0.000	**0.000**
	**16**	**Pipecolic Acid**	1.390	0.475	0.000	**0.000**
	**17**	**Lysine**	1.148	0.199	0.000	**0.000**
	**18**	**3-Hydroxy-3-Methylglutarate**	1.326	0.407	0.000	**0.000**
	**19**	**Ethylmalonic Acid**	1.267	0.342	0.000	**0.001**
	**20**	**4-Imidazoleacetic Acid**	0.631	-0.663	0.000	**0.001**
	**21**	**4-Pyridoxate**	1.494	0.579	0.000	**0.001**
	**22**	**N-Alpha-Acetyl-L-Asparagine**	1.356	0.440	0.000	**0.002**
	**23**	**Sn-Glycerol 3-Phosphate**	1.338	0.420	0.000	**0.002**
	**24**	**Alanine**	1.147	0.198	0.000	**0.002**
	**25**	**N-Acetylglycine**	0.753	-0.410	0.000	**0.002**
	**26**	**Succinic Acid**	1.548	0.631	0.000	**0.002**
	**27**	**Phosphocholine**	1.365	0.449	0.000	**0.003**
	**28**	**Glyoxylic Acid**	1.313	0.393	0.001	**0.003**
	**29**	**Thymine**	1.323	0.404	0.001	**0.004**
	**30**	**Pyruvic Acid**	1.153	0.205	0.001	**0.006**
	**31**	**3-Alpha,11-Beta,17-Alpha,21-Tetrahydroxy-5-Alpha-Pregnan-20-One**	1.583	0.662	0.001	**0.007**
	**32**	**Uracil**	1.380	0.465	0.002	**0.008**
	**33**	**Nepsilon,Nepsilon,Nepsilon-Trimethyllysine**	1.228	0.297	0.002	**0.008**
	**34**	**3-(4-Hydroxyphenyl)Lactate**	1.410	0.496	0.002	**0.010**
	**35**	**Thymidine**	1.347	0.430	0.003	**0.012**
	**36**	**Carnosine**	1.824	0.867	0.003	**0.012**
	**37**	**Pyridoxal**	1.311	0.391	0.003	**0.014**
	**38**	**Formyl-L-Methionyl Peptide**	1.313	0.393	0.004	**0.016**
	**39**	**Fumaric Acid**	1.245	0.316	0.004	**0.016**
	**40**	**Uric Acid**	1.238	0.308	0.004	**0.017**
	**41**	**Malic Acid**	1.229	0.298	0.004	**0.017**
	**42**	**Arginine**	1.068	0.096	0.005	**0.017**
	**43**	**Betaine**	1.112	0.154	0.006	**0.022**
	**44**	**Glycerate**	1.293	0.371	0.007	**0.023**
	**45**	**Erythritol**	1.163	0.217	0.007	**0.023**
	**46**	**N-Acetyl-L-Alanine**	1.188	0.248	0.007	**0.024**
	**47**	**Carnitine**	1.182	0.241	0.008	**0.026**
	**48**	**Inosine**	0.452	-1.144	0.009	**0.027**
	**49**	**3-(4-Hydroxyphenyl)Pyruvate**	1.329	0.411	0.011	**0.034**
	**50**	**2-Hydroxybutyric Acid**	0.833	-0.263	0.012	**0.036**
	**51**	**Gluconic Acid**	1.351	0.434	0.013	**0.039**
	**52**	**Adipic Acid**	1.396	0.481	0.014	**0.039**
	**53**	**N(Pai)-Methyl-L-Histidine**	0.809	-0.306	0.013	**0.039**
	**54**	**Trigonelline**	1.558	0.640	0.014	**0.041**
	**55**	**Cysteine**	0.894	-0.162	0.015	**0.041**
	**56**	**Butanoate**	1.297	0.375	0.018	**0.048**
	**57**	**10-Hydroxydecanoate**	0.779	-0.360	0.018	**0.049**
HCR-CTRL-NONRUNNER	1	Pyridine-2,3-Dicarboxylate	0.379	-1.399	0.002	0.316
	2	Serine	0.817	-0.291	0.005	0.349

vs. LCR-CTRL-NONRUNNER	3	Trans-Cinnamate	8.357	3.063	0.012	0.357
	4	Indoxyl Sulfate	2.049	1.035	0.011	0.357
	5	Thromboxane B2	1.830	0.872	0.007	0.357
	6	N-Acetyl-L-Leucine	0.561	-0.834	0.017	0.424
	7	Hippurate	3.219	1.687	0.033	0.458
	8	Hydroxybenzoic Acid	0.633	-0.660	0.023	0.458
	9	Acetoacetate	0.636	-0.653	0.031	0.458
	10	N-Acetylglycine	0.755	-0.405	0.029	0.458
	11	Pyruvic Acid	1.136	0.184	0.030	0.458
	12	4-Imidazoleacetic Acid	0.580	-0.786	0.038	0.477
	13	Methyl Indole-3-Acetate	2.478	1.309	0.045	0.520
	14	N(Pai)-Methyl-L-Histidine	0.710	-0.495	0.048	0.520
	15	Threonine	0.705	-0.504	0.055	0.523
	16	Thiamine Monophosphate	0.743	-0.428	0.052	0.523
	17	Guanosine	0.061	-4.031	0.060	0.539
	18	N-Amidino-L-Aspartate	0.738	-0.439	0.068	0.571
	19	()11(12)-Epete	1.527	0.611	0.077	0.613
	20	Lysine	1.149	0.200	0.082	0.625

Table S3: Examining the effect of rat line on enriched metabolic pathways (MSEA). Twenty most significant metabolite sets of pooled HCR vs. LCR and HCR-CTRL-NONRUNNER vs. LCR-CTRL-NONRUNNER groups.

Comparison	Number	Enriched metabolite sets	Total Compounds	Hits	p-palue (unadjusted)	FDR-adjusted p-value
HCR vs. LCR (pooled)	**1**	**Galactose metabolism**	27	2	0.000	**0.000**
	**2**	**Ascorbate and aldarate metabolism**	9	1	0.000	**0.000**
	**3**	**Inositol phosphate metabolism**	30	1	0.000	**0.000**
	**4**	**Selenocompound metabolism**	20	1	0.000	**0.003**
	**5**	**beta-Alanine metabolism**	21	2	0.000	**0.003**
	**6**	**Lysine degradation**	30	1	0.002	**0.009**
	**7**	**Alanine, aspartate and glutamate metabolism**	28	2	0.002	**0.011**
	**8**	**Pantothenate and CoA biosynthesis**	20	3	0.003	**0.011**
	**9**	**Histidine metabolism**	16	1	0.003	**0.011**
	**10**	**Vitamin B6 metabolism**	9	1	0.003	**0.012**
	**11**	**Arginine biosynthesis**	14	1	0.005	**0.014**
	**12**	**Pyrimidine metabolism**	39	7	0.007	**0.018**
	**13**	**Thiamine metabolism**	7	1	0.015	**0.037**
	**14**	**Glutathione metabolism**	28	3	0.018	**0.042**
	15	Cysteine and methionine metabolism	33	2	0.027	0.060
	16	Arginine and proline metabolism	36	3	0.029	0.060
	17	Glycine, serine and threonine metabolism	33	6	0.045	0.087
	18	Purine metabolism	70	5	0.049	0.089
	19	Taurine and hypotaurine metabolism	8	3	0.081	0.141
	20	Amino sugar and nucleotide sugar metabolism	42	1	0.257	0.424
HCR-CTRL-NONRUNNER vs. LCR-CTRL-NONRUNNER	1	D-Amino acid metabolism	15	1	0.005	0.195
	2	Sphingolipid metabolism	32	2	0.008	0.195
	3	Nicotinate and nicotinamide metabolism	15	2	0.017	0.279
	4	Glycine, serine and threonine metabolism	33	11	0.027	0.302
	5	Glycolysis / Gluconeogenesis	26	1	0.030	0.302
	6	Cysteine and methionine metabolism	33	7	0.063	0.529
	7	Biotin metabolism	10	2	0.074	0.532
	8	Lipoic acid metabolism	28	3	0.096	0.573
	9	Tyrosine metabolism	42	6	0.111	0.573
	10	Glyoxylate and dicarboxylate metabolism	31	9	0.138	0.573
	11	Ubiquinone and other terpenoid-quinone biosynthesis	18	4	0.153	0.573
	12	Tryptophan metabolism	41	4	0.155	0.573
	13	Thiamine metabolism	7	2	0.160	0.573
	14	Lysine degradation	30	4	0.176	0.573
	15	Purine metabolism	70	9	0.202	0.573
	16	Amino sugar and nucleotide sugar metabolism	42	1	0.215	0.573
	17	Terpenoid backbone biosynthesis	18	1	0.225	0.573
	18	Pyruvate metabolism	23	3	0.229	0.573
	19	Galactose metabolism	27	2	0.243	0.573
	20	Ascorbate and aldarate metabolism	9	2	0.243	0.573

Table S4: Examining the effect of BCAA supplementation on metabolite levels. Twenty most significantly changed metabolites of pooled BCAA vs. CTRL, HCR-BCAA-NONRUNNER vs. HCR-CTRL-NONRUNNER, and LCR-BCAA-NONRUNNER vs. LCR-CTRL-NONRUNNER groups.

Comparison	Number	Metabolite	FC	log2(FC)	p-value (unadjusted)	FDR-adjusted p-value
BCAA vs. CTRL (pooled)	**1**	**Valine**	1.679	0.748	0.000	**0.000**
	**2**	**3-Hydroxybutanoic Acid**	0.704	-0.507	0.000	**0.002**
	**3**	**Glutamine**	1.102	0.140	0.000	**0.012**
	**4**	**Leucine**	1.860	0.895	0.001	**0.019**
	**5**	**N-Acetyl-L-Leucine**	3.400	1.766	0.001	**0.023**
	**6**	**Serine**	0.866	-0.207	0.001	**0.036**
	**7**	**Cytidine**	1.255	0.327	0.002	**0.048**
	8	Isoleucine	1.572	0.653	0.003	0.054
	9	Pipecolic Acid	0.797	-0.328	0.004	0.060
	10	Hypotaurine	0.832	-0.265	0.004	0.060
	11	Glycine	0.883	-0.180	0.005	0.068
	12	Aminoisobutanoate	0.711	-0.492	0.006	0.075
	13	Ophthalmic Acid (Glutathione/Gsh)	0.686	-0.544	0.031	0.303
	14	N-Amidino-L-Aspartate	0.820	-0.286	0.028	0.303
	15	Myo-Inositol	1.107	0.146	0.030	0.303
	16	Alanine	1.088	0.122	0.032	0.303
	17	4-Coumarate	0.378	-1.405	0.050	0.353
	18	4-Methyl-2-Oxo-Pentanoic Acid	1.348	0.431	0.049	0.353
	19	Methyl-2-Oxovaleric Acid	1.348	0.431	0.049	0.353
	20	Trigonelline	0.751	-0.414	0.049	0.353
HCR-BCAA-NONRUNNER vs. HCR-CTRL-NONRUNNER	1	N-Acetyl-L-Leucine	8.422	3.074	0.019	0.524
	2	4-Hydroxy-2-Quinolinecarboxylic Acid	5.001	2.322	0.027	0.524
	3	N-Acetyl-D-Tryptophan	3.146	1.653	0.056	0.524
	4	Leucine	2.446	1.291	0.035	0.524
	5	4-Hydroxyproline	2.381	1.251	0.076	0.524
	6	Isoleucine	2.001	1.000	0.045	0.524
	7	N-Acetyl-L-Phenylalanine	1.939	0.955	0.007	0.524
	8	Uridine	1.935	0.952	0.052	0.524
	9	Valine	1.846	0.884	0.042	0.524
	10	Thromboxane B2	1.648	0.720	0.059	0.524
	11	N-Acetylneuraminate	1.634	0.708	0.022	0.524
	12	Uracil	1.615	0.691	0.065	0.524
	13	Thiamine Monophosphate	1.533	0.616	0.060	0.524
	14	Homogentisate	1.519	0.603	0.049	0.524
	15	Formyl-L-Methionyl Peptide	1.466	0.551	0.053	0.524
	16	Cytidine	1.465	0.551	0.058	0.524
	17	Allantoin	1.435	0.521	0.039	0.524
	18	N-Alpha-Acetyl-L-Asparagine	1.363	0.447	0.071	0.524
	19	Nepsilon,Nepsilon,Nepsilon-Trimethyllysine	1.330	0.412	0.071	0.524
	20	Creatinine	1.233	0.303	0.073	0.524
LCR-BCAA-NONRUNNER vs. LCR-CTRL-NONRUNNER	1	Serine	0.744	-0.427	0.000	0.072
	2	Valine	1.852	0.889	0.009	0.346
	3	Threonine	0.633	-0.660	0.011	0.346
	4	N-Amidino-L-Aspartate	0.673	-0.572	0.020	0.346
	5	Aminoisobutanoate	0.683	-0.549	0.015	0.346
	6	Pipecolic Acid	0.690	-0.536	0.016	0.346
	7	N-Acetylglycine	0.743	-0.428	0.020	0.346
	8	3-Hydroxybutanoic Acid	0.748	-0.420	0.012	0.346
	9	Glycine	0.772	-0.372	0.013	0.346
	10	Ophthalmic Acid (Glutathione/Gsh)	0.504	-0.987	0.037	0.515
	11	Sarcosine	0.651	-0.620	0.036	0.515
	12	Glutamine	1.080	0.111	0.047	0.594
	13	4-Coumarate	0.156	-2.677	0.096	0.661
	14	N-Acetyl-L-Leucine	2.767	1.468	0.115	0.661
	15	Leucine	2.094	1.066	0.058	0.661
	16	Isoleucine	1.693	0.760	0.110	0.661
	17	3-(4-Hydroxyphenyl)Pyruvate	0.663	-0.592	0.072	0.661
	18	4-Methyl-2-Oxo-Pentanoic Acid	1.423	0.509	0.115	0.661
	19	Methyl-2-Oxovaleric Acid	1.423	0.509	0.115	0.661
	20	Adipic Acid	0.709	-0.497	0.070	0.661

Table S5: Examining the effect of BCAA supplementation on enriched metabolic pathways (MSEA). Twenty most significant metabolite sets of pooled BCAA vs. CTRL, HCR-BCAA-NONRUNNER vs. HCR-CTRL-NONRUNNER, and LCR-BCAA-NONRUNNER vs. LCR-CTRL-NONRUNNER groups.

Comparison	Number	Enriched metabolite sets	Total Compounds	Hits	p-palue (unadjusted)	FDR-adjusted p-value
BCAA vs. CTRL (pooled)	**1**	**Valine, leucine and isoleucine degradation**	40	1	0.000	**0.000**
	**2**	**Valine, leucine and isoleucine biosynthesis**	8	2	0.000	**0.000**
	**3**	**Pantothenate and CoA biosynthesis**	20	3	0.000	**0.001**
	**4**	**Glyoxylate and dicarboxylate metabolism**	32	1	0.005	**0.024**
	**5**	**Lipoic acid metabolism**	28	1	0.005	**0.024**
	**6**	**Porphyrin metabolism**	31	1	0.005	**0.024**
	**7**	**Glutathione metabolism**	28	3	0.005	**0.024**
	8	Primary bile acid biosynthesis	46	2	0.021	0.080
	9	Glycine, serine and threonine metabolism	33	6	0.022	0.080
	10	Ascorbate and aldarate metabolism	9	1	0.030	0.088
	11	Inositol phosphate metabolism	30	1	0.030	0.088
	12	Selenocompound metabolism	20	1	0.032	0.088
	13	Taurine and hypotaurine metabolism	8	3	0.038	0.096
	14	Pyrimidine metabolism	39	7	0.063	0.149
	15	Galactose metabolism	27	2	0.084	0.185
	16	Alanine, aspartate and glutamate metabolism	28	2	0.107	0.209
	17	beta-Alanine metabolism	21	2	0.107	0.209
	18	Amino sugar and nucleotide sugar metabolism	42	1	0.225	0.413
	19	Lysine degradation	30	1	0.257	0.446
	20	Fatty acid biosynthesis	47	1	0.392	0.647
HCR-BCAA-NONRUNNER vs. HCR-CTRL-NONRUNNER	**1**	Amino sugar and nucleotide sugar metabolism	42	1	0.022	0.693
	**2**	Pyrimidine metabolism	39	8	0.073	0.693
	**3**	Pentose phosphate pathway	23	2	0.086	0.693
	**4**	Valine, leucine and isoleucine biosynthesis	8	6	0.099	0.693
	**5**	Valine, leucine and isoleucine degradation	39	7	0.100	0.693
	**6**	Pantothenate and CoA biosynthesis	20	5	0.108	0.693
	**7**	Purine metabolism	70	8	0.108	0.693
	8	Nitrogen metabolism	6	2	0.139	0.693
	9	Thiamine metabolism	7	2	0.180	0.693
	10	Arginine biosynthesis	14	9	0.210	0.693
	11	Galactose metabolism	27	2	0.214	0.693
	12	Glyoxylate and dicarboxylate metabolism	31	9	0.226	0.693
	13	Selenocompound metabolism	20	1	0.231	0.693
	14	beta-Alanine metabolism	21	3	0.235	0.693
	15	Vitamin B6 metabolism	9	2	0.245	0.693
	16	Citrate cycle (TCA cycle)	20	6	0.247	0.693
	17	Glycerolipid metabolism	16	2	0.253	0.693
	18	Lysine degradation	30	4	0.256	0.693
	19	D-Amino acid metabolism	15	1	0.295	0.693
	20	Ubiquinone and other terpenoid-quinone biosynthesis	18	4	0.307	0.693
LCR-BCAA-NONRUNNER vs. LCR-CTRL-NONRUNNER	**1**	**D-Amino acid metabolism**	**15**	**1**	**0.000**	**0.024**
	**2**	Sphingolipid metabolism	32	2	0.003	0.067
	**3**	Glycine, serine and threonine metabolism	33	11	0.005	0.082
	**4**	Glyoxylate and dicarboxylate metabolism	31	9	0.018	0.213
	**5**	Valine, leucine and isoleucine biosynthesis	8	6	0.021	0.213
	**6**	Lipoic acid metabolism	28	3	0.026	0.218
	**7**	Valine, leucine and isoleucine degradation	39	7	0.035	0.247
	8	Porphyrin metabolism	31	2	0.043	0.271
	9	Cysteine and methionine metabolism	33	7	0.120	0.539
	10	Nitrogen metabolism	6	2	0.122	0.539
	11	Glycolysis / Gluconeogenesis	26	1	0.122	0.539
	12	Phenylalanine, tyrosine and tryptophan biosynthesis	4	3	0.134	0.539
	13	Ubiquinone and other terpenoid-quinone biosynthesis	18	4	0.144	0.539
	14	Glutathione metabolism	28	7	0.151	0.539
	15	Vitamin B6 metabolism	9	2	0.170	0.559
	16	Tyrosine metabolism	42	6	0.182	0.559
	17	Lysine degradation	30	4	0.190	0.559
	18	Phenylalanine metabolism	8	2	0.247	0.638

19	Butanoate metabolism	15	7	0.253	0.638
20	Pantothenate and CoA biosynthesis	20	5	0.258	0.638

Table S6: Examining the effect of voluntary running on metabolite levels. Twenty most significantly changed metabolites of pooled RUNNER vs. NONRUNNER, HCR-CTRL-RUNNER vs. HCR-CTRL-NONRUNNER, and LCR-CTRL-RUNNER vs. LCR-CTRL-NONRUNNER groups.

Comparison	Number	Metabolite	FC	log2(FC)	p-value (unadjusted)	FDR-adjusted p-value
RUNNER vs. NONRUNNER (pooled)	**1**	**4-Methyl-2-Oxo-Pentanoic Acid**	0.622	-0.686	0.000	**0.015**
	**2**	**Methyl-2-Oxovaleric Acid**	0.622	-0.686	0.000	**0.015**
	3	Omega-Hydroxydodecanoic Acid	0.742	-0.431	0.001	0.068
	4	Aminoisobutanoate	0.715	-0.485	0.002	0.084
	5	Lauric Acid	0.867	-0.206	0.003	0.084
	6	10-Hydroxydecanoate	0.777	-0.364	0.004	0.095
	7	Trigonelline	1.778	0.830	0.005	0.102
	8	Pyridine-2,3-Dicarboxylate	0.660	-0.600	0.005	0.102
	9	4-Guanidinobutanoate	1.883	0.913	0.008	0.116
	10	Succinic Acid	1.374	0.458	0.007	0.116
	11	Fumaric Acid	1.198	0.261	0.011	0.148
	12	Palmitoleic Acid	0.685	-0.547	0.021	0.160
	13	Myristic Acid	0.724	-0.466	0.015	0.160
	14	Gamma-Linolenic Acid	0.737	-0.440	0.022	0.160
	15	Creatine Phosphate	0.758	-0.400	0.023	0.160
	16	Phosphocholine	1.319	0.399	0.023	0.160
	17	Glutamic Acid	1.202	0.265	0.016	0.160
	18	Malic Acid	1.181	0.239	0.014	0.160
	19	3-Hydroxy-3-Methylglutarate	1.180	0.238	0.019	0.160
	20	Guanidinoacetate	1.166	0.222	0.022	0.160
HCR-CTRL-RUNNER vs. HCR-CTRL-NONRUNNER	1	Trigonelline	2.581	1.368	0.007	0.154
	2	Homogentisate	1.976	0.983	0.013	0.154
	3	3-Alpha,11-Beta,17-Alpha,21-Tetrahydroxy- 5-Alpha-Pregnan-20-One	1.873	0.905	0.013	0.154
	4	4-Methyl-2-Oxo-Pentanoic Acid	0.538	-0.896	0.011	0.154
	5	Methyl-2-Oxovaleric Acid	0.538	-0.896	0.011	0.154
	6	Thiamine Monophosphate	1.688	0.755	0.005	0.154
	7	Anthranilate	1.616	0.693	0.011	0.154
	8	5-Oxo-Proline	1.563	0.644	0.010	0.154
	9	Phosphocholine	1.548	0.630	0.012	0.154
	10	Uracil	1.547	0.629	0.007	0.154
	11	Galactitol	1.469	0.555	0.005	0.154
	12	Erythritol	1.384	0.469	0.007	0.154
	13	Deoxycarnitine	1.229	0.297	0.007	0.154
	14	N-Amidino-L-Aspartate	1.446	0.532	0.015	0.160
	15	Gamma-Linolenic Acid	0.548	-0.868	0.017	0.172
	16	Uridine	1.892	0.920	0.019	0.182
	17	4-Hydroxy-2-Quinolinecarboxylic Acid	4.035	2.013	0.023	0.201
	18	Creatine Phosphate	0.423	-1.242	0.027	0.201
	19	Guanidinoacetate	1.364	0.448	0.028	0.201
	20	Lauric Acid	0.764	-0.389	0.024	0.201
LCR-CTRL-RUNNER vs. LCR-CTRL-NONRUNNER	1	Serine	0.812	-0.301	0.002	0.295
	2	Threonine	0.682	-0.552	0.024	1.000
	3	3-(4-Hydroxyphenyl)Pyruvate	0.589	-0.763	0.026	1.000
	4	Proline	0.910	-0.136	0.034	1.000
	5	Taurochenodeoxycholic Acid	0.359	-1.477	0.049	1.000
	6	Asparagine	0.876	-0.191	0.055	1.000
	7	Carnosine	0.537	-0.897	0.057	1.000
	8	Pyridine-2,3-Dicarboxylate	0.591	-0.758	0.059	1.000
	9	Alanine	0.891	-0.167	0.064	1.000
	10	Creatine Phosphate	0.772	-0.373	0.074	1.000
	11	Gluconic Acid	0.759	-0.398	0.081	1.000
	12	N-Acetyl-Dl-Glutamic Acid	0.737	-0.441	0.097	1.000
	13	4-Methyl-2-Oxo-Pentanoic Acid	0.782	-0.355	0.116	1.000
	14	Methyl-2-Oxovaleric Acid	0.782	-0.355	0.116	1.000
	15	Indoxyl Sulfate	0.769	-0.379	0.120	1.000
	16	Glycocholate	0.573	-0.804	0.150	1.000
	17	Aminoisobutanoate	0.835	-0.261	0.164	1.000
	18	Diacetyl	1.175	0.232	0.165	1.000
	19	Pyridoxal	0.869	-0.203	0.171	1.000
	20	Ophthalmic Acid (Glutathione/Gsh)	0.630	-0.667	0.174	1.000

Table S7: Examining the effect of voluntary running on enriched metabolic pathways (MSEA). Twenty most significant metabolite sets of pooled RUNNER vs. NONRUNNER, HCR-CTRL-RUNNER vs. HCR-CTRL-NONRUNNER, and LCR-CTRL-RUNNER vs. LCR-CTRL-NONRUNNER groups.

Comparison	Number	Enriched metabolite sets	Total Compounds	Hits	p-palue (unadjusted)	FDR-adjusted p-value
RUNNER vs. NONRUNNER (pooled)	1	Valine, leucine and isoleucine degradation	39	7	0.001	0.062
	2	Valine, leucine and isoleucine biosynthesis	8	6	0.005	0.112
	3	Nicotinate and nicotinamide metabolism	15	2	0.008	0.112
	4	Pyruvate metabolism	23	3	0.010	0.112
	5	Arginine and proline metabolism	36	13	0.016	0.112
	6	Butanoate metabolism	15	7	0.016	0.112
	7	Galactose metabolism	27	2	0.020	0.112
	8	Citrate cycle (TCA cycle)	20	6	0.020	0.112
	9	Fatty acid biosynthesis	47	4	0.020	0.112
	10	Steroid hormone biosynthesis	87	1	0.028	0.133
	11	Tyrosine metabolism	42	6	0.029	0.133
	12	Propanoate metabolism	21	2	0.035	0.145
	13	Glyoxylate and dicarboxylate metabolism	31	9	0.040	0.155
	14	Porphyrin metabolism	31	2	0.056	0.189
	15	Alanine, aspartate and glutamate metabolism	28	11	0.057	0.189
	16	Nitrogen metabolism	6	2	0.062	0.193
	17	Glycerophospholipid metabolism	36	3	0.080	0.225
	18	Glycine, serine and threonine metabolism	33	11	0.083	0.225
	19	Histidine metabolism	16	4	0.085	0.225
	20	beta-Alanine metabolism	21	3	0.100	0.243
HCR-CTRL-RUNNER vs. HCR-CTRL-NONRUNNER	1	Thiamine metabolism	7	2	0.007	0.166
	2	Lysine degradation	30	4	0.010	0.166
	3	Galactose metabolism	27	2	0.011	0.166
	4	Steroid hormone biosynthesis	87	1	0.013	0.166
	5	Vitamin B6 metabolism	9	2	0.029	0.214
	6	Valine, leucine and isoleucine degradation	39	7	0.031	0.214
	7	Pantothenate and CoA biosynthesis	20	5	0.034	0.214
	8	Terpenoid backbone biosynthesis	18	1	0.049	0.214
	9	beta-Alanine metabolism	21	3	0.053	0.214
	10	Tryptophan metabolism	41	4	0.058	0.214
	11	Butanoate metabolism	15	7	0.060	0.214
	12	Arginine and proline metabolism	36	13	0.061	0.214
	13	Linoleic acid metabolism	5	1	0.066	0.214
	14	Fatty acid biosynthesis	47	4	0.066	0.214
	15	Biosynthesis of unsaturated fatty acids	36	3	0.068	0.214
	16	Glutathione metabolism	28	7	0.071	0.214
	17	Citrate cycle (TCA cycle)	20	6	0.076	0.214
	18	Tyrosine metabolism	42	6	0.079	0.214
	19	Valine, leucine and isoleucine biosynthesis	8	6	0.082	0.214
	20	Propanoate metabolism	21	2	0.086	0.214
LCR-CTRL-RUNNER vs. LCR-CTRL-NONRUNNER	1	D-Amino acid metabolism	15	1	0.002	0.097
	2	Sphingolipid metabolism	32	2	0.006	0.142
	3	Selenocompound metabolism	20	1	0.064	0.791
	4	Phenylalanine, tyrosine and tryptophan biosynthesis	4	3	0.108	0.791
	5	Glycine, serine and threonine metabolism	33	11	0.111	0.791
	6	Cysteine and methionine metabolism	33	7	0.137	0.791
	7	Nicotinate and nicotinamide metabolism	15	2	0.146	0.791
	8	Valine, leucine and isoleucine biosynthesis	8	6	0.169	0.791
	9	Ubiquinone and other terpenoid-quinone biosynthesis	18	4	0.219	0.791
	10	Pentose phosphate pathway	23	2	0.222	0.791
	11	Tyrosine metabolism	42	6	0.235	0.791
	12	Glycolysis / Gluconeogenesis	26	1	0.240	0.791
	13	Primary bile acid biosynthesis	46	6	0.248	0.791
	14	beta-Alanine metabolism	21	3	0.258	0.791
	15	Glyoxylate and dicarboxylate metabolism	31	9	0.258	0.791
	16	Amino sugar and nucleotide sugar metabolism	42	1	0.272	0.791
	17	Biotin metabolism	10	2	0.299	0.791
	18	Vitamin B6 metabolism	9	2	0.329	0.791
	19	Alanine, aspartate and glutamate metabolism	28	11	0.335	0.791
	20	Histidine metabolism	16	4	0.340	0.791

Table S8: Examining the interactive effects of BCAA supplementation and voluntary running on metabolite levels. Twenty most significantly changed metabolites of pooled BCAA-RUNNER vs. CTRL-NONRUNNER, HCR-BCAA-RUNNER vs. HCR-CTRL-NONRUNNER, and LCR-BCAA-RUNNER vs. LCR-CTRL-NONRUNNER groups.

Comparison	Number	Metabolite	FC	log2(FC)	p-value (unadjusted)	FDR-adjusted p-value
BCAA-RUNNER vs. CTRL-NONRUNNER (pooled)	**1**	**Aminoisobutanoate**	0.459	-1.124	0.000	**0.000**
	**2**	**3-Hydroxybutanoic Acid**	0.566	-0.820	0.000	**0.003**
	**3**	**Cytidine**	1.397	0.482	0.000	**0.013**
	**4**	**Serine**	0.804	-0.315	0.001	**0.039**
	**5**	**Omega-Hydroxydodecanoic Acid**	0.727	-0.460	0.001	**0.044**
	**6**	**Ophthalmic Acid (Glutathione/Gsh)**	0.354	-1.500	0.002	**0.046**
	**7**	**Uracil**	1.551	0.633	0.002	**0.046**
	**8**	**Lauric Acid**	0.827	-0.274	0.002	**0.046**
	9	Succinic Acid	1.632	0.707	0.004	0.066
	10	10-Hydroxydecanoate	0.741	-0.433	0.004	0.066
	11	4-Guanidinobutanoate	2.361	1.239	0.005	0.068
	12	Valine	1.474	0.559	0.008	0.077
	13	Threonine	0.688	-0.539	0.007	0.077
	14	Ethylmalonic Acid	1.282	0.359	0.008	0.077
	15	Hypotaurine	0.783	-0.354	0.008	0.077
	16	Myo-Inositol	1.236	0.305	0.007	0.077
	17	N-Acetylglycine	0.713	-0.489	0.009	0.083
	18	Pyridine-2,3-Dicarboxylate	0.491	-1.028	0.011	0.093
	19	3-Hydroxy-3-Methylglutarate	1.303	0.382	0.012	0.095
	20	Acetoacetate	0.616	-0.700	0.013	0.098
HCR-BCAA-RUNNER vs. HCR-CTRL-NONRUNNER	**1**	**Aminoisobutanoate**	0.372	-1.425	0.000	**0.018**
	**2**	**Uracil**	1.993	0.995	0.000	**0.018**
	**3**	**Myo-Inositol**	1.357	0.440	0.001	**0.029**
	**4**	**Omega-Hydroxydodecanoic Acid**	0.649	-0.624	0.001	**0.033**
	**5**	**Cytidine**	1.544	0.626	0.001	**0.035**
	**6**	**N-Alpha-Acetyl-L-Asparagine**	1.566	0.647	0.001	**0.036**
	**7**	**Gamma-Linolenic Acid**	0.498	-1.005	0.002	**0.037**
	**8**	**10-Hydroxydecanoate**	0.625	-0.678	0.002	**0.037**
	**9**	**Galactitol**	1.443	0.529	0.002	**0.037**
	**10**	**Succinic Acid**	2.011	1.008	0.002	**0.037**
	**11**	**Lauric Acid**	0.755	-0.405	0.003	**0.037**
	**12**	**Palmitoleic Acid**	0.437	-1.193	0.004	**0.049**
	13	Phosphocholine	1.622	0.697	0.006	0.066
	14	Uridine	1.931	0.949	0.006	0.068
	15	3-Hydroxybutanoic Acid	0.493	-1.020	0.009	0.079
	16	Erythritol	1.383	0.468	0.009	0.079
	17	Deoxycarnitine	1.368	0.452	0.008	0.079
	18	Linoleate	0.494	-1.018	0.011	0.081
	19	Valine	1.921	0.942	0.011	0.081
	20	Myristic Acid	0.553	-0.855	0.011	0.081
LCR-BCAA-RUNNER vs. LCR-CTRL-NONRUNNER	**1**	**Serine**	0.769	-0.378	0.000	**0.006**
	**2**	**Aminoisobutanoate**	0.597	-0.743	0.001	**0.037**
	**3**	**3-Hydroxybutanoic Acid**	0.700	-0.514	0.001	**0.037**
	**4**	**Hypotaurine**	0.724	-0.466	0.001	**0.037**
	5	N-Amidino-L-Aspartate	0.642	-0.639	0.003	0.084
	6	Pipecolic Acid	0.690	-0.536	0.011	0.273
	7	Ophthalmic Acid (Glutathione/Gsh)	0.431	-1.214	0.013	0.280
	8	Threonine	0.651	-0.619	0.015	0.280
	9	Pyridine-2,3-Dicarboxylate	0.525	-0.929	0.019	0.318
	10	Indoxyl Sulfate	0.680	-0.555	0.034	0.524
	11	Cytidine	1.340	0.423	0.043	0.594
	12	3-(4-Hydroxyphenyl)Pyruvate	0.662	-0.595	0.073	0.740
	13	Erythritol	0.820	-0.286	0.059	0.740
	14	N-Acetylglycine	0.821	-0.285	0.069	0.740
	15	Cysteic Acid	0.837	-0.256	0.067	0.740
	16	4-Coumarate	0.283	-1.820	0.123	0.807
	17	Deoxycorticosterone Acetate	1.789	0.839	0.113	0.807
	18	Indole-3-Acetic Acid	0.631	-0.663	0.106	0.807
	19	N-Acetyl-L-Phenylalanine	1.474	0.559	0.199	0.807
	20	4-Guanidinobutanoate	1.403	0.489	0.183	0.807

Table S9: Examining the effect of BCAA supplementation and voluntary running on enriched metabolic pathways (MSEA). Twenty most significant metabolite sets of pooled BCAA-RUNNER vs. CTRL-NONRUNNER, HCR-BCAA-RUNNER vs. HCR-CTRL-NONRUNNER, and LCR-BCAA-RUNNER vs. LCR-CTRL-NONRUNNER groups.

Comparison	Number	Enriched metabolite sets	Total Compounds	Hits	p-palue (unadjusted)	FDR-adjusted p-value
BCAA-RUNNER vs. CTRL-NONRUNNER (pooled)	**1**	**Valine, leucine and isoleucine degradation**	39	7	0.000	**0.012**
	**2**	**D-Amino acid metabolism**	15	1	0.001	**0.026**
	**3**	**Glycine, serine and threonine metabolism**	33	11	0.003	**0.043**
	**4**	**Butanoate metabolism**	15	7	0.004	**0.043**
	**5**	**Galactose metabolism**	27	2	0.004	**0.043**
	6	Sphingolipid metabolism	32	2	0.006	0.053
	7	Valine, leucine and isoleucine biosynthesis	8	6	0.008	0.056
	8	Pyrimidine metabolism	39	8	0.009	0.059
	9	Glyoxylate and dicarboxylate metabolism	31	9	0.015	0.073
	10	Tyrosine metabolism	42	6	0.016	0.073
	11	Propanoate metabolism	21	2	0.017	0.073
	12	Pyruvate metabolism	23	3	0.018	0.073
	13	Pantothenate and CoA biosynthesis	20	5	0.019	0.073
	14	Nicotinate and nicotinamide metabolism	15	2	0.024	0.084
	15	Ascorbate and aldarate metabolism	9	2	0.033	0.098
	16	Inositol phosphate metabolism	30	2	0.033	0.098
	17	Citrate cycle (TCA cycle)	20	6	0.033	0.098
	18	Glutathione metabolism	28	7	0.037	0.103
	19	Selenocompound metabolism	20	1	0.041	0.103
	20	Alanine, aspartate and glutamate metabolism	28	11	0.041	0.103
HCR-BCAA-RUNNER vs. HCR-CTRL-NONRUNNER	**1**	**Galactose metabolism**	27	2	0.000	**0.002**
	**2**	**Ascorbate and aldarate metabolism**	9	1	0.001	**0.006**
	**3**	**Inositol phosphate metabolism**	30	1	0.001	**0.006**
	**4**	**Pantothenate and CoA biosynthesis**	20	3	0.001	**0.006**
	**5**	**Fatty acid biosynthesis**	47	1	0.003	**0.018**
	**6**	**beta-Alanine metabolism**	21	2	0.003	**0.019**
	**7**	**Pyrimidine metabolism**	39	7	0.005	**0.023**
	**8**	**Valine, leucine and isoleucine degradation**	40	1	0.011	**0.046**
	9	Vitamin B6 metabolism	9	1	0.023	0.078
	10	Valine, leucine and isoleucine biosynthesis	8	2	0.024	0.078
	11	Lysine degradation	30	1	0.039	0.110
	12	Selenocompound metabolism	20	1	0.040	0.110
	13	Alanine, aspartate and glutamate metabolism	28	2	0.045	0.111
	14	Tryptophan metabolism	41	1	0.047	0.111
	15	Purine metabolism	70	5	0.051	0.113
	16	Arginine and proline metabolism	36	3	0.095	0.195
	17	Amino sugar and nucleotide sugar metabolism	42	1	0.112	0.218
	18	Taurine and hypotaurine metabolism	8	3	0.181	0.320
	19	Arginine biosynthesis	14	1	0.184	0.320
	20	Histidine metabolism	16	1	0.220	0.347
LCR-BCAA-RUNNER vs. LCR-CTRL-NONRUNNER	1	Taurine and hypotaurine metabolism	8	3	0.009	0.168
	2	Glutathione metabolism	28	3	0.010	0.168
	3	Valine, leucine and isoleucine biosynthesis	8	2	0.020	0.224
	4	Glycine, serine and threonine metabolism	33	6	0.076	0.395
	5	Primary bile acid biosynthesis	46	2	0.080	0.395
	6	Glyoxylate and dicarboxylate metabolism	32	1	0.096	0.395
	7	Lipoic acid metabolism	28	1	0.096	0.395
	8	Porphyrin metabolism	31	1	0.096	0.395
	9	Fatty acid biosynthesis	47	1	0.123	0.448
	10	Valine, leucine and isoleucine degradation	40	1	0.146	0.448
	11	Ascorbate and aldarate metabolism	9	1	0.163	0.448
	12	Inositol phosphate metabolism	30	1	0.163	0.448
	13	Pantothenate and CoA biosynthesis	20	3	0.205	0.521
	14	Thiamine metabolism	7	1	0.250	0.589
	15	Cysteine and methionine metabolism	33	2	0.311	0.615
	16	Histidine metabolism	16	1	0.313	0.615
	17	beta-Alanine metabolism	21	2	0.337	0.615
	18	Lysine degradation	30	1	0.344	0.615
	19	Pyrimidine metabolism	39	7	0.354	0.615

20	Galactose metabolism	27	2	0.386	0.637
